# Glutamine Synthetase 1 Increases Autophagy Lysosomal Degradation of Mutant Huntingtin Aggregates in Neurons, Ameliorating Motility in a *Drosophila* Model for Huntington’s Disease

**DOI:** 10.3390/cells9010196

**Published:** 2020-01-13

**Authors:** Luisa Vernizzi, Chiara Paiardi, Giusimaria Licata, Teresa Vitali, Stefania Santarelli, Martino Raneli, Vera Manelli, Manuela Rizzetto, Mariarosa Gioria, Maria E. Pasini, Daniela Grifoni, Maria A. Vanoni, Cinzia Gellera, Franco Taroni, Paola Bellosta

**Affiliations:** 1 Department of Biosciences, University of Milan, 20133 Milan, Italy; luisa.vernizzi@imls.uzh.ch (L.V.); chiara.paiardi@gmail.com (C.P.); vitaliteresa@yahoo.it (T.V.); martino.raneli@gmail.com (M.R.); manellivera@gmail.com (V.M.); mariarosa.gioria@unimi.it (M.G.); maria.pasini@unimi.it (M.E.P.); maria.vanoni@unimi.it (M.A.V.); 2 Department of Cellular, Computational and Integrative Biology (CiBio), University of Trento, 38123 Trento, Italy; stefania.santarelli@studenti.unitn.it; 3 Unit of Medical Genetics and Neurogenetics, Fondazione IRCCS Istituto Neurologico Carlo Besta, 20133 Milan, Italy; manurizzetto@yahoo.it (M.R.); Cinzia.Gellera@istituto-besta.it (C.G.); Franco.Taroni@istituto-besta.it (F.T.); 4 Department of Pharmacy and Biotechnology, University of Bologna, 40126 Bologna, Italy; daniela.grifoni@unibo.it; 5 Department of Medicine, NYU Langone Medical Center, New York, NY 10016, USA

**Keywords:** Huntington’s disease, glutamine synthetase 1, autophagy, TOR signaling, protein aggregates, *Drosophila* model for neuronal degeneration

## Abstract

Glutamine Synthetase 1 (GS1) is a key enzyme that catalyzes the ATP-dependent synthesis of l-glutamine from l-glutamate and is also member of the Glutamate Glutamine Cycle, a complex physiological process between glia and neurons that controls glutamate homeostasis and is often found compromised in neurodegenerative diseases including Huntington’s disease (HD). Here we report that the expression of GS1 in neurons ameliorates the motility defects induced by the expression of the mutant Htt, using a *Drosophila* model for HD. This phenotype is associated with the ability of GS1 to favor the autophagy that we associate with the presence of reduced Htt toxic protein aggregates in neurons expressing mutant Htt. Expression of GS1 prevents the TOR activation and phosphorylation of S6K, a mechanism that we associate with the reduced levels of essential amino acids, particularly of arginine and asparagine important for TOR activation. This study reveals a novel function for GS1 to ameliorate neuronal survival by changing amino acids’ levels that induce a “starvation-like” condition responsible to induce autophagy. The identification of novel targets that inhibit TOR in neurons is of particular interest for the beneficial role that autophagy has in preserving physiological neuronal health and in the mechanisms that eliminate the formation of toxic aggregates in proteinopathies.

## 1. Introduction

Huntington’s disease (HD) is an inherited neurodegenerative disease with a middle age clinical onset that highly depends upon the length of the CAG repeated sequence (>35) present in the first exon of the *huntingtin* gene (OMIM 143100) [1]. Mutations in the gene that expands this sequence result in a protein with a long poly-Q trait that forms toxic mHTT protein aggregates which are considered as one of the major cause for the progressive degeneration of neurons, particularly of the striatum and cortex, resulting in cognitive decline and motor defects [2,3,4]. Drugs as antisense oligonucleotides [5] have been recently developed to slow down the disease progression, and attention is dedicated to those that ameliorate neuronal survival by increasing autophagy to limit the formation of mHTT aggregates [6,7,8,9,10].

Neuronal health depends also upon maintaining glutamate at physiological levels; a process that is controlled by a sequence of biochemical reactions, called the Glutamate–Glutamine Cycle (GGC), occurring between glia and neurons that often are found altered in neuronal pathology [11]. Key components of the GGC are the enzymes Glutamine Synthetase-1 (GS1) that uses ammonia to convert glutamate into glutamine with the hydrolysis of ATP, Glutamate dehydrogenase (GDH), that coverts glutamate into alfa-keto glutarate (aKG), and Glutaminase (GLS) that in neurons produces glutamate from glutamine [12]. Since the activity of GS1 was found reduced in neuronal diseases [13] and in the postmortem brains of patients with HD [14,15,16], we decided to investigate the contribution of GS1 to HD, using a well-established *Drosophila* model for HD that expresses the exon1 of the human *HTT* gene with 93 CAG repeats, (hereon referred to as *Htt-Q93*). This model recapitulates the cellular and molecular events described in HD, including the loss of neurons and impaired animal motility [17].

Gs1 is a conserved enzyme, from bacteria to vertebrates, and in *Drosophila* there are two distinct genes, *Gs-1* and *Gs-2*, encoding for *glutamine synthetases,* both highly homologous to the human gene *GLUL* (60.5%) [18]. Here we show that the expression in neurons of *GS1* together with *Htt-Q93* significantly improves animal motility and rescues neuronal loss. At the cellular level, we found that *GS1* increases the level of autophagy, and significantly reduces the size of Htt-Q93 protein aggregates.

Autophagy is induced when amino acid levels are low, and in neurons it plays an important role for the survival and homeostasis of these post-mitotic cells, while its activation is counteracted by TOR signaling and nutrients [19]. Activation of TOR by amino acids induces the assembling of the RagA/B-C/D GTPases complex that, together with the GTPase Rheb, activates the TORC1 complex at the lysosomal membrane [20,21] to phosphorylate S6K and 4EBP target proteins [22]. Interestingly, we found that the expression of GS1 in neurons was able to reduce TOR signaling, measured by the reduced level of S6K phosphorylation, a mechanism that was present also when GS1 was co-expressed with Htt-Q93. Analysis of the amino acid levels in the heads of animals expressing GS1 in neurons reveals a significant decrease of essential amino acids, including proline and arginine, known to be necessary in the mechanism of TOR activation. Finally, we show that GS1 protein levels are reduced in human fibroblasts from HD patients, and these cells have impairment in the autophagy flux, suggesting that the role of GS1 in the control autophagy may be conserved also in human cells.

In summary, our data propose a novel function for GS1 in neurons that links its activity to mechanisms that activates autophagy and the reduction of Htt-Q93 toxic aggregates. Understanding how GS1 controls amino acids signaling in neurons is the initial step to comprehend a novel function for this enzyme, member of the GGC, in the control of autophagy and neuronal survival. Ultimately, this would be relevant not only for the control of pathological diseases such as HD, but also in the mechanisms that regulate physiological neuronal health and aging.

## 2. Materials and Methods

### 2.1. Fly Husbandry and Lines

Animals were raised at low density, at 25 °C, on a standard food medium containing 9 g/L agar (ZN5 B and V, Gatattico (Re), Italy), 75 g/L corn flour, 60 g/L white sugar, 30 g/L brewers’ yeast (Fisher Scientific Italia, Rodano (Mi), Italy), 50 g/L fresh yeast and 50 mL/L molasses (Biosigma, Cona (Ve), Italy), along with nipagin and propionic acid (Fisher, Rodano (Mi), Italy). The following fly lines were obtained from the Bloomington *Drosophila* Stock Center: *UAS-Htt-16Q* (B33810), *EP-GS1(G33347)* an enhancer-promoter (EP)-line with an insertion of the *P*-element, carrying a *UAS* enhancer sequence and a basal promoter, in the *GS1* locus allowing the expression of *GS1* by Gal4 (B27940), *UAS-GS1-RNAi* (B40836), *UAS-mCherry-Atg8a* (B37750), *elav^c155^-Gal4* on the *X ch.* (B458) and *GMR-Gal4* (B9146). Fly lines bearing the *UAS-Htt-Q93* was a gift of from L. Marsh (University of California, Irvine), *UAS-GFP-LAMP1* and *UAS-Atg1-RNAi* where a gift from F. Parisi (Beatson Institute for Cancer Research, Glasgow, UK), *UAS-Atg5-RNAi* was a gift from T. Neufeld (University of Minnesota). *EP-FOXO* [23] was a gift from Hugo Stocker (ETH-Zurich).

### 2.2. Survival at Eclosure Analysis

The number of animals and their timing of eclosion from every cross with the *elav^c155^-Gal4* driver were quantified, and the percentage of animals eclosed was calculated for each of the genotypes of interest with respect to the expected number of animals that did eclose.

### 2.3. Motility Assays

Ten larvae (3rd instar) were collected, washed with phosphate-buffered saline (PBS) and then transferred to a 14 cm Petri dish containing 1% agarose in PBS, where a visible grid was drawn to portray the arena (Supplementary Figure S4). The number of lines crossed by one larva in 1 min at room temperature was counted. Statistical analysis was performed with the Student’s *t*-test; values for each genotype are represented as mean ± standard deviation (SD). Experiments were repeated at least three times. For motility assays with adult animals, 10–15 females were collected at 1 day after eclosion (DAE), transferred in a plastic vial, and their ability to climb up the empty vial after a knock-down to the bottom was analyzed, using the protocol previously described in Zhang et al., [24]. The number of flies that were able to climb half of the tube in 15 s was counted. Values were expressed as the percentage of success with respect to the total number of flies in the vial. For each genotype the test was repeated 30 times for each time point. After the test, adults were transferred in vials with food, and these vials were changed every two days. Data are represented as a curve of progressive motility impairment. The statistical analysis of variance (one-way analysis of variance (ANOVA) was performed using PRISM 7 GraphPad Software (San Diego, CA, USA). Every experiment was repeated at least six times. The experiments were performed only using females, as in males the expression of *Htt-Q93* resulted in a low survival rate, and *elav/Y*; *Htt-Q93*/+ males showed 50% reduction of their climbing activity at 2–3 DAE, and that were not further used (Supplementary Figure S6). The stronger effect of *Htt-Q93* in males is probably due to the dosage compensation that affects genes expressed on the *X* chromosome where the transgene *elav-Gal4* is inserted [25].

### 2.4. Quantification of Fluorescence (GFP) on the Adult Compound Eye

Photographs of adult eyes expressing the indicated UAS-transgenes in the retina, and co-expressing *UAS-GFP* using the *GMR-Gal4* promoter, were taken at 8 days after eclosion using a Leica stereomicroscope MZ10F with a fluorescent source and a camera, and by employing 5× magnification. The intensity of fluorescence in each image was quantified as the integrated density from an area containing 20 ommatidia using Adobe Photoshop-CS4, and expressed as a GFP intensity/area. At least 10 eyes (one for each animal) were used for each genotype, and these experiments were repeated twice.

### 2.5. Western Blot

Proteins were extracted from ten larval brains or from ten adult heads, collected in lysis buffer (50 mM Hepes/NaOH pH 7.4, 250 mM NaCl, 1 mM ethylenediaminetetraacetic acid (EDTA), 1.5% Triton X-100 containing a cocktail of phosphatases inhibitors (PhosSTOP #04906837001, Merck Life Science, Darmstadt, Germany) and proteases inhibitors (Roche, cOmplete #04693132001, Merck Life Science, Darmstadt, Germany). For the detection of the huntingtin protein, lysates were extracted using 2% sodium dodecyl sulfate (SDS) lysis buffer (50 mM Hepes/NaOH pH 7.4, 250 mM NaCl, 1 mM EDTA, 2% SDS containing phosphatases and proteases inhibitors). Samples were sonicated three times for 10 s using a Branson Ultrasonic Sonifier 250 (Branson, Darbury, CA, USA) equipped with a microtip set at 25% power. Tissue and cell debris were removed by centrifugation at 10,000× *g* for 30 min at 4 °C. Proteins in the crude extract were quantified by a bicinchoninic acid (BCA) Protein assay Reagent Kit (Pierce), following the manufacturer’s instructions with bovine serum albumin as the standard protein. For SDS-PAGE, samples were incubated for 8 min at 100 °C in standard reducing loading buffer; 40 µg of total protein were run on a SDS-polyacrylamide gel and transferred onto nitrocellulose membranes (GE-Healthcare, Fisher Scientific Italia) After blocking in 5% (*w*/*v*) non-fat milk in tris-buffered saline (TBS)-0.05% Tween (TBS-T), membranes were incubated overnight with primary antibodies against: hHTT (1:500, ab109115), GABARAB (1:500, ab109364) that cross-reacts with *Drosophila* Atg8a [26,27], GS1 (1:800, ab64613) from Abcam, Cambridge, UK; Elav (1:100, #9F8A9), Synapsin (1:100, #8C3), Actin5c (1:200, #JL20) from Developmental Studies Hybridoma Bank (DSHB), University of Iowa, IA, USA. SQSTM1/p62 (1:500, #5114), *Drosophila* p70-S6K (1:500, #9209), ATG1 (1:500, #8054) from Cell Signaling, Euroclone (Mi) Italy; Tubulin (1:500, MAB3408) from Chemicon, DBA Italia, Segrate (Mi), Italy; and ATG5 (1:500, OSA00026W) from Thermo Fisher Monza, Monza, Italy.

### 2.6. Filter Trap Assay

Ten larval brains or ten adult heads for each genotype were homogenized in lysis buffer (50 mM Tris- HCl pH 6.8, 2% SDS plus proteases and phosphatases inhibitors). Fifty micrograms of protein extracts were loaded on a dot–blot device and filtered through a cellulose acetate membrane (0.22 μm pore size), previously washed in 1% (*w*/*v*) SDS solution in PBS. After filtration, membranes were washed three times in TBS-T, saturated in 5% non-fat milk and processed for immunodetection with anti-Htt antibodies from Abcam, as described above.

### 2.7. Patient-Derived Cell Lines

Fibroblasts from patient and controls were derived from skin biopsies and grown in the Dulbecco’s modified Eagle’s medium (DMEM)-high glucose or galactose media, as described previously in Wong et al., [28]. Each individual providing a biological sample signed a written, informed consent approved by the Institutional Review Board of the Fondazione IRCCS Istituto Neurologico Carlo Besta, Milan, Italy, in agreement with the Declaration of Helsinki.

### 2.8. MS/HPLC for Glutamate and Glutamine Quantification and Analysis of Amino Acids

Ten heads from animals at eight DAE were resuspended in 100 μL of Hepes/NaOH with proteases and phosphatases inhibitors, samples were disrupted using a mechanical tip following two rounds of sonication of 10 s each. After centrifugation at 13,000 rpm for 30 min at 4 °C, 20 μL of each sample were set aside for Bradford analysis, while supernatants were diluted in buffer containing DL-Lysine D4 and DL-Ornitine D6 as internal standards (Eurisotop, Cambridge Isotopes Labs, Inc., Cambridge, UK) and 2/3 *v*/*v* of methanol, to precipitate the proteins. After centrifugation, amino acid analysis was performed on a Perkin Elmer Series 200 HPLC equipped with a 4 μm, 2.0× 75 mm Synergi Polar-RP (Phenomenex, Bologna, Italy) column, and coupled with triple quadrupole API2000 tandem mass spectrometer and AB SCIEX analyst software (version 1.4) for data acquisition analysis (AB SCIEX, Framingham, MA, USA). Analysis of free amino acids was performed by Innovhub, Stazioni Sperimentali per Industria, Milan (Italy) with the following protocol: 30 heads of each genotype were resuspended in 300 μL of HPLC-grade H_2_O, samples were disrupted using a mechanical tip following two rounds of sonication of 10 s each.

20 μL were set aside for Bradford analysis, while 75 μL of a 6% solution of sulfosalicylic acid were added to the final concentration of 1.5%. Samples were centrifuged for 20 min at 13,000 rpm, and supernatants were used for the analysis. The protocol applied (UNI 22615:1992) uses ionic exchange chromatography where the amino acids are separated by chromatography using an automatic Lithium High Performance Physiological Column with the instrument Biochrom 30+ from Biochrom. The separation is based on using Lithium Citrated buffer solutions employing a five steps pH separation from pH 2.80 up to pH 3.55. Amino acids were visualized on TLC using ninidrin and detected at 440 and 570 nm.

### 2.9. Quantitative Real Time PCR

Total RNA was isolated using the RNeasy Mini Kit (Qiagen Italia, Milano, Italy) according to the manufacturer’s instructions. Extracted RNAs were quantified using an ultraviolet (UV) spectrophotometer, and RNA integrity was confirmed with ethidium bromide staining. The purified RNA (1 mg) was used as the template for cDNA using SuperScript II (Invitrogen, San Diego, CA, USA, Thermo Fisher, Waltham, MA, USA). The SYBR Green PCR Kit (Qiagen) was used, and products were quantified utilizing the BIO-RAD system. The quantitative reverse transcription polymerase chain reaction (qRT-PCR) sample values were normalized for the expression of *actin-5C* mRNA. The relative level for each gene was calculated using the 2-DDCt method [29] and reported as arbitrary units. Three separate samples were used in triplicate. Primers sequences are reported in Parisi et al., [30].

### 2.10. Quantification of GS1 Activity

*Drosophila* larvae or heads that had been flash frozen in liquid nitrogen were resuspended in 200 μL 50 mM Hepes/NaOH buffer, pH 7.4 and 0.5% protease inhibitors cocktail (Roche, cOmplete **#**04693132001 Merck, Milano, Italy). They were homogenized with a Branson 250 sonifier equipped with a microtip (25% power, 20% duty cycle) by applying four series of 10 sonication pulses and controlling the temperature by immersion in an ice bath. The homogenate was centrifuged in a microfuge at 13,000 rpm for 30 min at 4 °C and passed through an Ultrafree-MC (Millipore UFC306V, Merck, Milano, Italy) filter device by centrifuging at 11,000 rpm for 15 min at 4 °C. The filtrate was used for protein and activity assays. Protein concentration was determined with the Bradford method and bovine serum albumin as the reference protein. Glutamine Synthetase (GS) activity was assayed by quantifying the amount of γ-glutamylhydroxyamate (GGHA) produced as described in Vorhaben et al., Caizzi et al., and De Pinto et al., [31,32,33]. In preliminary experiments a range of GGHA concentrations (0.07–0.15 mM) was used to determine the sensitivity and linear range of the assay. Briefly, a known amount of crude extract (0.1–0.5 mg in up to 120 μL) was incubated for 1 h at 37 °C in 50 mM Tris/HCl buffer, pH 7.5, hydroxylamine (40 mM final concentration), MgCl2 (40 mM) L-glutamate (20 mM) and ATP (5 mM) in a final volume of 600 μL. The reaction was blocked by adding 200 μL of FeCl_3_ solution (3.3% FeCl_3_, 8% trichloroacetic acid (TCA), 0.67 M HCl) and centrifuged at top speed for 10 min. The amount of GGHA produced was calculated from the absorbance value at 500 nm and 600 nm with the HP 8453 diode array spectrophotometer using the reported extinction coefficients of 1.004 mM^−1^cm^−1^ and 0.471 mM^−1^ cm^−1^ for the crude extracts derived from larvae and heads, respectively; by monitoring the entire spectrum of the final solution, we ensured that no artifacts arose from turbidity, precipitation or the presence of pigments, especially with *Drosophila* heads. For each assay a blank devoid of L-Glu and ATP was set up, and the measured absorbance was subtracted to that of the corresponding sample prior to the calculation of the activity value. The extracts were selected in order to ensure that the GGHA concentration increase was linear with respect to the incubation time. l-Methionine sulfoxide (MSO) was added to reaction mixtures at a final concentration of 1 mM (from 10 mM stock solution in 50 mM Hepes/NaOH buffer, pH 7.4) to test its effect on GS1 activity. Activity was expressed as milliunits/mg protein, where 1 unit of GS activity is the amount of GS that converts 1 μmol l-Glu into product per minute.

GS activity detected in wild-type larvae was taken as 100% activity to normalize the values obtained with different larvae batches and in comparison with the values measured with transfected larvae. For each sample, replicates differed by less than 15%. GS1-specific activity in the heads of adult females was analyzed in the presence of the specific GS1-inhibitor methionine sulfoximine (MSO), as shown in Supplementary Figure S7.

### 2.11. Immunofluorescences and Quantification of the Size of Htt-Q93 Aggregates

Brains from third instar larvae were dissected and fixed for 30 min in 4% paraformaldehyde (PFA) on ice. After tissues permeabilization with 0.5% Triton X-100, samples were washed in PBS-0.1% Tween20 (PBST) and blocked in 3% BSA (in PBST) for 30 min on ice. Samples were incubated with anti-Htt antibody in 3% BSA overnight at 4 °C. After three washes of 10 min each in PBST, the samples were incubated with mouse or rabbit secondary antibodies Alexa 555 (Invitrogen) 1:400 in 3% BSA for 2 h and at room temperature (RT) on the shaker. After extensive washing with PBST, brains were mounted on slides in 20 mL of Mowiol. Nuclei were stained with Hoechst 33258 added at the final concentration of 1µg/mL. The quantification of the mHtt aggregates visualized by fluorescence upon staining with anti-human Htt antibodies and secondary anti-rabbit Alexa 555, was performed on *z*-stacks acquired under a confocal microscope, by keeping the acquisition parameters constant. The intensity of the red aggregates was quantified from the confocal stacks using ImageJ 1.50i software (Wayne Rasband NIH, Bethesda, MA, USA) inside a square of fixed size (300 × 300 pixels), which was moved in four different regions in the specific area of the brain (calyx, VNC or IL). The aggregates were quantified by measuring the intensity of the fluorescent signal in these different regions (integrated density-IntDen tool on ImageJ). The integrated density of the fluorescence acquired was considered directly proportional to the aggregate number. Statistics was applied using as a threshold the IntDen in the control animals.

### 2.12. Statistical Analysis

Student *t*-test analysis and analysis of the variance calculated using One-way ANOVA with Tukey multi-comparisons test (the whole ANOVA analysis is reported in the Supplementary Excel 1) were calculated using GraphPad-PRISM 7. *p* values are indicated with asterisks * *p* < 0.05, ** *p* < 0.01, *** *p* < 0.001, **** *p* < 0.0001, respectively.

### 2.13. Autophagy Analysis

*elav-Gal4*; *UAS-GFP*; *UAS-mCherry-Atg8a* females were crossed with males carrying the appropriate UAS-transgenes. Third instar larvae (72–80 h after hatching) were collected and washed, and then their brains were dissected in phosphate-buffered saline (PBS) buffer. Brains were fixed for 30 min in 4% paraformaldehyde (PFA) on ice. Nuclei were stained with Hoechst 33258, samples mounted in Mowiol and images acquired using a confocal microscope (SM5 LEICA Leserteknik GmbH, Wetzlar, Germany) using ImageJ 1.50i software or *AdobePhotoshop-CS4.* The quantification of autophagic puncta stained by mCherry-Atg8a and representing the autophagosomes, was performed on *z*-stacks acquired under a +confocal microscope, by keeping the acquisition parameters constant. The intensity of the autophagic puncta was quantified with the software *ImageJ* inside a square of fixed size (300 × 300 pixels), which was moved in four different regions in the specific area of the brain (calyx, VNC or retina). Autophagy was quantified by measuring the intensity of the fluorescent signal in these different regions (integrated density-IntDen tool on ImageJ) using four out of five brains for each genotype of interest. Statistics was applied using as a threshold the IntDen in the control animals

## 3. Results

### 3.1. GS1 Rescues Neuronal Death Induced by Expression of Htt-Q93 in the Retina

**Figure 1:**
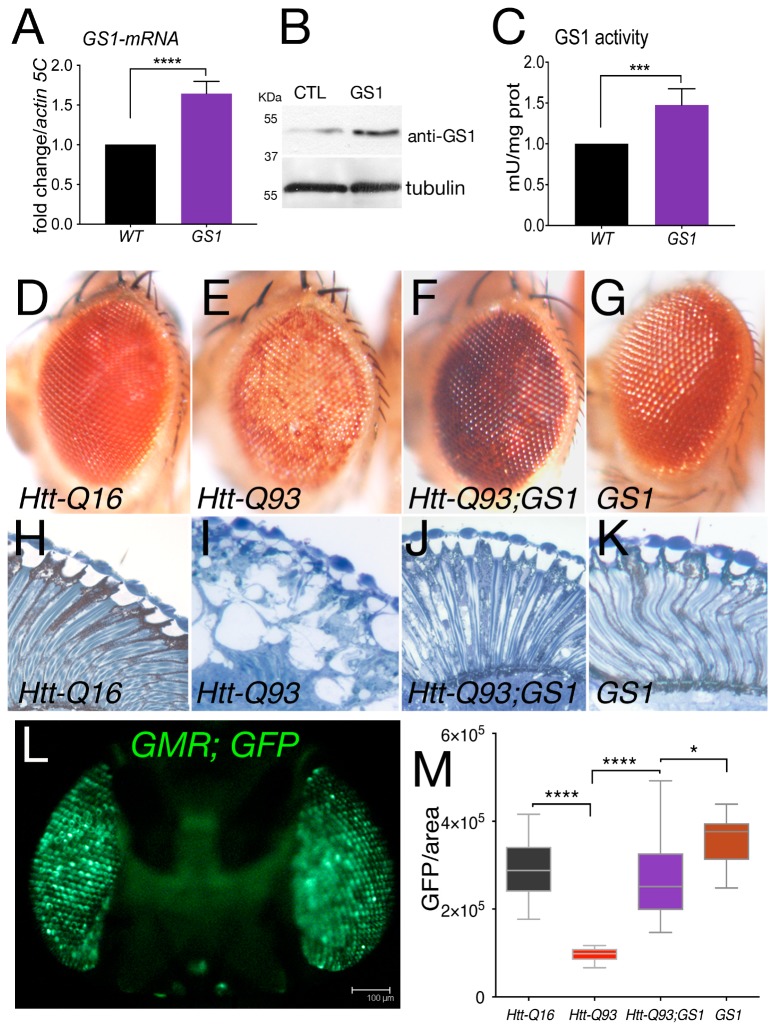
Expression of *GS1* in the retina rescues neuronal death induced by *Htt-Q93*. (**A**) Quantitative reverse transcription polymerase chain reaction (qRT-PCR) analysis of *GS1 mRNA* expression in larvae from control *w^1118^* of animals expressing the *EP-GS1^G4337^ (GS1)* line using the *actin-Gal4* driver. The relative level of *GS1-mRNA* is reported as arbitrary units compared to *actin5C* used as a control. At least three separate experiments were performed in duplicate. (**B**) Western blot from larvae extracts showing the relative amount of GS1 protein from *w^1118^* (CTL) animals or expressing GS1. Tubulin was used as the loading control. (**C**) GS1 enzymatic activity in extracts from whole larvae used in (**A**,**B**). *** *p* < 0.001, **** *p* < 0.0001 in panels (**A**,**C**) were calculated from Student’s *t*-test from at least three independent experiments.(**D**–**G**)) Photographs of *Drosophila*-compound eyes (lateral view) from females at 20 days after eclosion (DAE) expressing the indicated *UAS*-transgenes using the *GMR-Gal4* driver. (**H**–**K**) Representative photographs of the retina from animals of the indicated genotype. (**L**) Fluorescent image of a fly head expressing *UAS-GFP* under the *GMR-Gal4* promoter (sagittal view), bar 100 μm. (**M**) Quantification of GFP from photographs of adult eyes at 20 DAE of the indicated genotypes. The asterisks represent the *p*-values from One-way analysis of variance (ANOVA) with Tukey multiple comparison * *p* < 0.05 and **** *p* < 0.0001, and the error bars indicate the standard deviations.

In post mortem brains from HD patients, the expression of the enzyme glutamine synthetase (GS1) was found reduced. In order to evaluate the potential role of GS1 in HD we expressed the *Drosophila*, homolog of the human *GLUL* gene (Supplementary Figure S1), in neurons of the retina using a well-established model for HD. These animals expressed *exon1*-of the human *huntingtin* gene containing 93 repetitions of the CAG sequence, herein called *Htt-Q93* and were shown to recapitulate phenotypes of the disease including neuronal loss and motility defects [34,35]. As shown in Figure 1, ubiquitous expression of *Drosophila* GS1, using the *EP-GS1^-G3347^* line, hereon called *UAS-GS1*, resulted in a twofold increase in *Gs1-mRNA* (Figure 1A) and in a 50 kDa protein recognized by anti-human GS1 antibodies (Figure 1B). Analysis of GS1 enzymatic activity showed a significant increase compared to that in control *w^1118^* animals (Figure 1C), suggesting that the *EP-GS1^-G3347^* line is expressing an active *Drosophila* GS1 enzyme.

When *UAS-GS1* was co-expressed with *UAS-Htt-Q93* in the retina using the *GMR-Gal4* promoter, we observed a complete rescue of the reduced pigmentation visible in the ommatidia of Htt-Q93 animals (compare Figure 1E with Figure 1F). On the contrary, reducing *GS1* expression together with *Htt-Q93* exacerbated these defects, and animals showed a premature loss of pigment cells and eye morphology defects, in that at 8 days after eclosion, it was similar to that of animals expressing *UAS-Htt-Q93* at 20 days (Supplementary Figure S2). An ultramicroscopic analysis showed that expression of GS1 ameliorated also the alteration of the cornea morphology induced by *Htt-Q93* expression and visualized by the presence of large vacuoles indicating cell loss (Figure 1I,J). Morphological analysis of the retina from *UAS-GS1* and control *UAS-Htt-Q16* animals revealed no defects (Figure 1H,K, respectively).

Neuronal death can be indirectly analyzed by measuring the decrease over time of the fluorescence emitted by the Green Fluorescent Protein (GFP) when co-expressed with Htt-Q93 [35] (Figure 1L). These experiments showed that co-expression of *GS1* completely rescued the reduction in GFP induced by *Htt-Q93* (Figure 1M, ANOVA *p* < 0.0001), while we did not observe any significant difference in the quantification of GFP in the ommatidia of control *GMR-Q16* or *GMR-GS1* animals. A representative image for each genotype is shown in Supplementary Figure S3.

### 3.2. GS1 Expression in Neurons Improves Locomotion Defects of Htt-Q93 Animals

**Figure 2:**
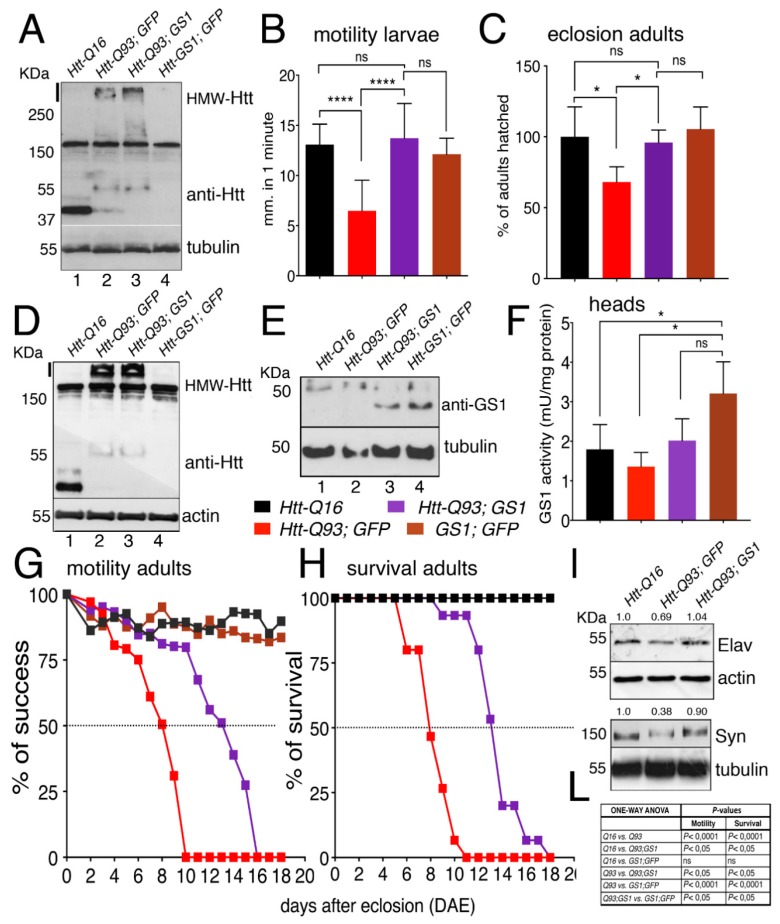
Expression of GS1 in neurons rescues motility defects and neuronal death induced by Htt-Q93. (**A**) Western blot showing the relative amounts of the protein huntingtin in lysates from 20 third instar larval brains expressing the indicated transgenes using the pan-neuronal driver elav-Gal4. Tubulin was used as the loading control. The band of about 30 KDa in lane 1 represents the Htt-Q16 protein, while the band at 55 KDa, in lanes 2 and 3, represents the Htt-Q93 protein. The high molecular weight proteins recognized by the anti-hHTT antibody also present in lanes 2 and 3 represent the mutant Htt high form that jammed in the stacking gel (indicated by a black line on the side). The 150 KDa band present in all the lanes is aspecific, since it is recognize by the mHtt antibody also in lane 4 where Htt is not expressed. A similar description is applied to the Western in Figure 2D. (**B**) Quantification of the motility in third instar larvae of the indicated genotype, where the data are expressed as the distance traveled in one minute measured in mm, and 10 larvae from each genotype were used the experiment was repeated three times (see Supplementary Figure S4C–E, movies S1 and S2). (**C**) Eclosion timing of adult females of the relative genotypes. (**D**,**E**) Western blots showing the respective amounts of huntingtin and GS1 proteins in lysates from 15 heads for each genotype, of females at 8 days after eclosion (DAE), wherein actin and tubulin were used as a loading control, respectively. In 2E is visible a band of about 30–40 KDa only when the *UAS-GS1* transgene is expressed (lane 3 and 4). The band at the higher molecular weight of about 55 KDa is either the endogenous *Drosophila* GS1 that is recognized by the anti-human GS1 antibody, or an aspecific band, since it is present in all the lanes also where GS1 is not overexpressed. (**F**) GS1 enzymatic activity (mUnits/mg of total proteins) in lysates from heads of females at 8 DAE, data show the significant increase of GS1 activity (brown) in these animals compared to that in the heads of un-transfected control Q16 animals (black bar), and from Htt-Q93 (red) or Htt-Q93 co-expressing GS1 (purple). The * = *p* < 0.05 values in (**F**) were calculated from Student’s *t*-test from at least three independent experiments, and error bars indicate the standard deviations. (**G**) Motility assay on adult females, where data are expressed as % of success to the motility in control animals. (**H**) Analysis of adult lethality is expressed as the % of survival of females relative to control. The ability of GS1 to ameliorate the motility of *Htt-Q93* was confirmed using three independent *Htt-Q93, GS1* recombinant lines (Supplementary Figure S8). (**I**) Western blot showing the amounts of endogenous neuronal specific proteins elav and synapsin in lysates extracted from fifteen heads of the indicated genotype, actin and tubulin were used as control, and the % of reduction of the relative protein is calculated compared to that in control animals where is considered equal to 1. (**L**) Table reporting ANOVA for the data in panels G and H. The **** *p* < 0.0001 and * *p* < 0.05 values in panels (**B**,**C**,**F**) were calculated using one-way ANOVA with Tukey multiple comparison from at least three independent experiments, and error bars indicate the standard deviations.

We next extended our analysis to all neurons using the *elav^c155^-Gal4* pan-neuronal promoter (hereon referred as *elav-Gal4*) [34]. Western blot analysis on extracts from larval brains from *elav*-*Htt-Q16* and *elav*-*Htt-Q93* animals showed the presence of a 35 kDa and 55 kDa proteins, respectively, recognized by anti-human HTT antibodies. In addition, a high-molecular-weight protein-complex > 250 kDa (HMW-mHtt) was visibly entrapped in the stacking gel, where extracts from *elav-Htt-Q93* animals were loaded, and almost no reduction in the HMW-mHtt was detected when *Htt-Q93* was co-expressed with *GS1* (Figure 2A), suggesting that GS1 was only partially able to reduce the high molecular weight form of HMW-mHtt aggregates at this stage of expression.

Since one characteristic of HD is the progressive impairment of movement [34], we investigated whether expression of *UAS-GS1* could rescue the motility defects in third instar larvae expressing *UAS-Htt-Q93* in neurons by performing motility assays. These experiments showed that the expression of *GS1* was able to fully rescue these defects that in *elav-Htt-Q93* larvae are already visible at 48–72 h after egg laying (AEL) (Figure 2B, ANOVA *p* < 0.0001 and Supplementary Figure S4 and Supplementary Video S1 and S2). In addition, *UAS-GS1* also rescued the developmental lethality visible in *elav-Htt-Q93* animals (Figure 2C, ANOVA *p* < 0.05). No defects in morphology or motility were observed in larvae expressing *UAS-GS1* or *UAS-Htt-Q16*.

Western blot analysis on proteins extracted from the heads of adult females showed a pattern of expression for Htt-Q16 and Htt-Q93 that was similar to that found in the larval brains (Figure 2D). In addition, GS1 protein was expressed with a similar amount in the lysates from animals expressing *UAS-Htt-Q93* together with *UAS-GS1* or *UAS-GS1* alone (Figure 1E). Analysis of the relative GS1 enzymatic activity showed a similar increased in extracts from animal expressing *UAS-GS1* alone and those expressing *UAS-GS1* together with *UAS-Htt-Q93* indicating that GS1 was expressed with a similar activity in both genotypes (Figure 2F, ANOVA *p* < 0.05). Finally, we analyzed the climbing activity on adult females (Supplementary Figure S4 and Supplementary Video S3). This data showed that *elav/+; Htt-Q93* animals presented a 50% reduction in their motility at 8 days after eclosion (Figure 2G,L, ANOVA *p* < 0.0001), that was extended up to 14 days by expression of *GS1* (Figure 2G, ANOVA *p* < 0.05); a similar effect for GS1 was observed in the rescue of animal survival (Figure 2H, ANOVA *p* < 0.05 and panel L). Flies expressing *GS1* alone or *Htt-Q16* did not show any visible alterations in their motility or viability. 

We then analyzed indirectly, the ability of GS1 to rescue neuronal death by quantifying in western blot the expression of the neural proteins Elav and Synapsin (Figure 2I) in the adult heads. This data showed that, co-expression of *GS1* rescued the reduced level of Elav (69%) and of Synapsin (38%) proteins detected in *Htt-Q93* corroborating its function on neuronal survival.

### 3.3. Expression of GS1 Reduces Htt-Q93 Protein Aggregates in Neurons

**Figure 3:**
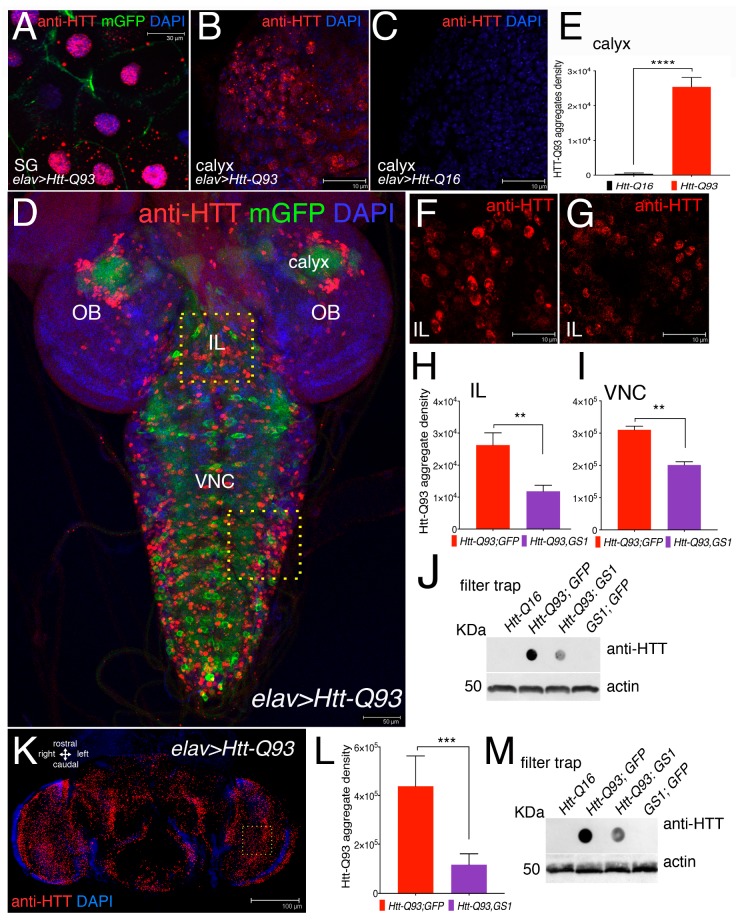
Expression of GS1 in neurons reduces the presence of Htt-Q93 aggregates. (**A**) Confocal microscopy images of cells from the salivary gland (SG), where the *elav-Gal4* insertion is also transcriptionally active, showing the presence of Htt-Q93 protein aggregates (red), that are visible in the cytoplasm and in the nuclei of cells expressing *UAS-Htt-Q93* and *UAS-GFP*, immunostained with anti-hHtt antibodies. Nuclei are stained with 4′,6-diamidino-2-phenylindole (DAPI); bar 30 µm. (**B**) Htt-Q93 aggregates visualized in the region from the calyx, (**C**) that are not detected in the calyx from control *Htt-Q16* animals; bar 10 µm; (**E**) relative quantification of the fluorescence in (**B**,**C**). (**D**) Confocal image of the whole brain from third instar larvae expressing *Htt-Q93* and *GFP* (*elav-Gal4*; *Htt-Q93*; *mGFP*); the Htt-Q93 aggregates are visualized by immunofluorescence and nuclei with DAPI, bar 50 µm. The yellow squares represent the regions where the Htt-Q93 aggregates were quantified: the Ventral Nerve Cord (VNC) and the intralobe region (IL), in addition to the calyx. (**F**,**G**) Picture representing the Htt-Q93 aggregates visualized in the intralobe (IL) region from *Htt-Q93* (F) or *Htt-Q93; GS1* (**G**) animals; bar10 µm; the relative quantification analysis is shown from the IL (**H**) and in the Ventral Nerve Cord (VNC) (**I**). Quantification of the aggregate density was calculated in the relative area and measured as the integrated density of the immunofluorescence signal visualized using anti-human Htt antibody from 10 confocal *z*-stacks for each animal and elaborated with ImageJ, (see Materials and Methods). (**J**) Filter trap analysis using lysates from the brains of the indicated transgenes; the expression of actin in the same extracts was analyzed by western blot as an internal control for loading. (**K**) Confocal image of *elav-Gal4; Htt-Q93* adult female’s head at 10 DAE, immunostained with anti-hHtt antibody showing the presence of Htt-Q93 aggregates (RED, nuclei DAPI), bar 100 µm. (**L**) Quantification of the Htt-Q93 aggregates in the heads of the animals of the indicated genotypes from confocal *z*-stacks; analysis was performed in the area indicated by the square. (**M**) Filter trap analysis of Htt-Q93 protein aggregates using lysates from the heads. Actin was used as the internal control. The ** *p* < 0.01, *** *p* < 0.001, **** *p* < 0.0001, values in panels (**E**,**I**,**J**,**M**) were calculated from Student’s *t*-test from at least three independent experiments, where error bars indicate the standard deviations. OL = optic lobe.

Mutant huntingtin protein forms toxic intracellular aggregates that largely contribute to neuronal death. Using the *elav-Gal4* promoter, that is transcriptionally active in neurons and in the gigantic cells of the salivary glands, we expressed *UAS-Htt-Q93* together with membrane *UAS-GFP* and used an anti-human Htt antibody to detect the presence of Htt-Q93 protein aggregates (mHtt) by immunofluorescence (Figure 3A–D,F,G).

At 72 h of development, mHtt-aggregates were clearly visible in the cytoplasm and in the nuclei of the cells of the salivary gland (SG) (Figure 3A) and in the cells of the calyx of Htt-Q93 animals (Figure 3B), but absent in cells of the calyx from Htt-Q16 larvae (Figure 3C). The ability of GS1 to modify the formation of Htt Q93 aggregates was analyzed by quantifying mHTT immunofluorescence in neurons from the Intra-Lobe (IL), and from the Ventral Nerve Cord (VNC) (Figure 3F). This analysis was performed using the signal for the integrated density of the fluorescence acquired from *z*-stacks using a defined threshold that considers the number and size of the aggregates. This analysis showed that co-expression of *GS1* with *Htt-Q93* significantly reduced the number of mHtt aggregates, in neurons from the IL (Figure 3G,H, *t*-student, *p* < 0.01) and from the VNC (Figure 3I, *t*-student, *p* < 0.01). To support these results we performed a filter-trap assay, a technique that is used to trap large protein aggregates on a cellulose acetate membrane where they can be visualized by immunoblot. These experiments showed that the co-expression of *GS1* reduced the amount of large Htt-Q93 aggregates trapped on the membrane, while no Htt-Q93 aggregates were detected in extracts from the brains of animals expressing *UAS-Htt-Q16* or *UAS-GS1* alone (Figure 3J). Similar experiments were performed in adult animals using extracts from females’ heads at 10 DAE. These experiments indicate that GS1 significantly reduces the presence of Htt-Q93 aggregates (Figure 3M, integrated analysis in analysis 3J, *t*-student, *p* < 0.001), suggesting that its ability to control the formation of the toxic Htt-Q93 aggregates is conserved though development.

### 3.4. GS1 Induces Autophagy in Neurons

**Figure 4:**
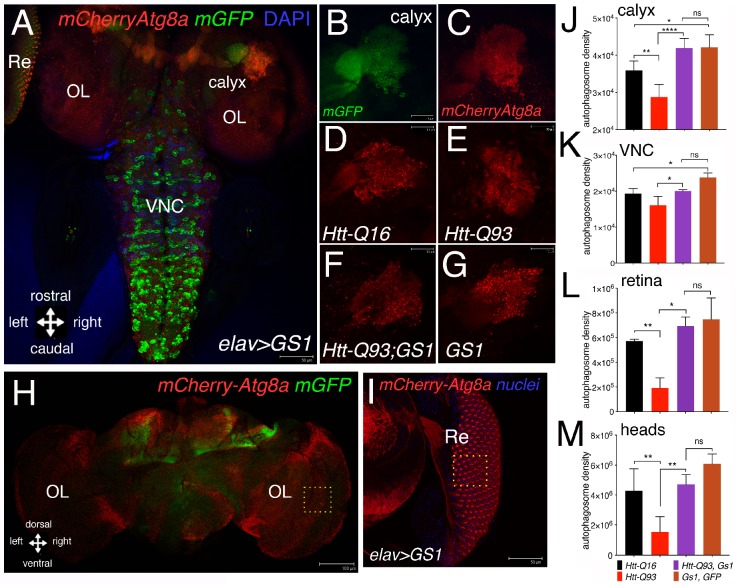
Expression of GS1 in neurons induces autophagy. (**A**) Confocal image of whole the brain from a third instar larva showing the pattern of expression for *elav-mCherryAtg8a; GFP* where the autophagic puncta are marked in red and neurons by GFP; the images represent the pattern of expression of *elav-mCherryAtg8a* from animals expressing *UAS-GS1*. (**B**,**C**) Insets showing a higher magnification of the right calyx from A; neurons are visualized by *elav-GFP.* (**B**) and autophagic puncta by *elav*-*mCherryAtg8a* (**C**); bar 10 µm. Autophagic puncta were analyzed in the region of the calyx (**D–G**), retina (Re) (**I**) and in the Ventral Nerve Cord (VNC); OL = optic lobe. (**D**–**G**) representative images showing *mCherryAtg8a* staining in the calyx of the animals at the indicated genotypes, (a higher magnification of these images is shown in Supplementary Figure S10). (**H**) Image of an *elav-mCherry-Atg8a; mGFP* female’s head at 10 DAE; OL = optic lobe; bar 100 µm. (**I**) Image of an eye disc from a third instar *elav-GS1* larva co-expressing *mCherry*-Atg8a in neurons of the retina and in the pigmented cells, with the nuclei stained in blue; bar 50 µm. (**J**–**M**) Quantification of autophagy was measured as the integrated density of *mCherry-Atg8a* and then converted in autophagosome density (see methods). Autophagosome density in neurons of the calyx (**J**), in the Ventral Nerve Cord (VNC), (**K**) in the retina (**L**), and in the adult heads (**M**) from animals of the indicated genotypes. One-way ANOVA *p*-values analysis: **** *p* < 0.0001, ** *p* < 0.01, * *p* < 0.05 were calculated from a minimum of five animals, error bars indicate the standard deviations.

Neurons employ autophagy to efficiently clear cellular debris that are detrimental for their homeostasis, including toxic protein aggregates [36]. To investigate whether GS1 may activate the autophagy–lysosomal pathway, we analyzed whether or not its expression induces the formation of the characteristic autophagosome vesicles, visible as autophagic puncta and marked by the ubiquitin-like protein Atg8a, the homolog of the human LC3, that localize at the autophagosome [37]. We used Atg8a fused with the mCherry-reporter [38] that was co-expressed with mGFP in neurons using the *elav-Gal4* promoter. Fluorescence associated to mCherry-Atg8a was analyzed in the third instar larvae, specifically in the neurons of the calyx (Figure 4A,D–G), the Ventral Neuronal Cord (VNC) (Figure 4A), and the retina (Re) (Figure 4A,I). Quantification of this analysis showed that the expression of *UAS-GS1* was able to significantly rescue the reduction in the number of autophagic puncta induced by *UAS-HttQ93* in all the regions analyzed (Figure 4J,K,L), in addition its expression induced the formation of autophagosomes particularly significant in cells of the calyx and of the VNC (Figure 4J,K). A similar analysis was performed in adults, where the integrated density of the autophagic puncta was quantified in female’ brains at 10 DAE (Figure 4H). These data are consistent with those from larval brains, and showed that the co-expression of *GS1* with *Htt-Q93*, resulted in a significant rescue of the autophagic puncta (Figure 4M; ANOVA *p* < 0.01). No significant differences were found in the number of autophagosomes between *UAS-Htt-Q93*, *GS1* and *UAS-GS1* animals (ANOVA *p* = ns).

### 3.5. GS1 Rescue of Htt-Q93 Motility-Defect Depends on the Full Expression of Atg1 and Atg5

**Figure 5:**
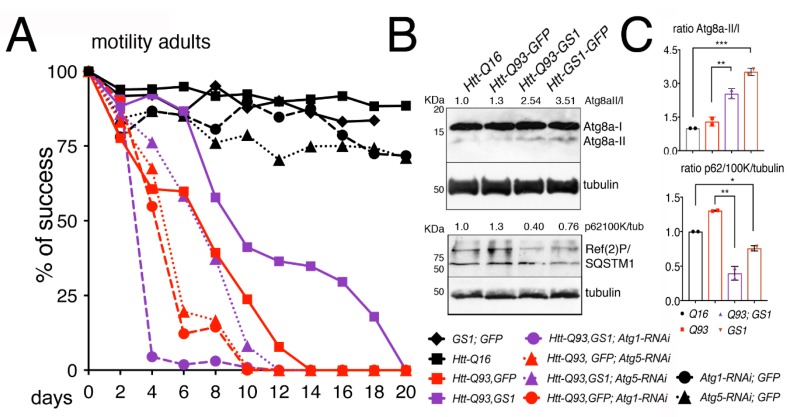
Reduction of *Atg1* and *Atg5* significantly impairs the ability of GS1 to rescue the motility defects induced by *Htt-Q93*. (**A**) 15 adult females were used for each genotype and their % of success in motility reported in the graph, the *p*-values from the one-way ANOVA are reported in the Excel file 1. (**B**) The presence of GS1 favors Atg8a cleavage and reduces the presence of Ref(2)P. Western blots from heads of females at 10 DAE of the indicated genotype, showing the pattern of expression of Atg8a-I and II and of SQSTM1/Ref(2)P, note that in *Drosophila* the latter runs in two isoforms of about 100 and 62 KDa, both recognized by the Drosophila antibody. Tubulin was used as the control for loading. Experiments were repeated at least twice with similar results. 10 heads were used for each genotype. (**C**) The ratio for Atg8a-II/I and Ref(2)P/tubulin were calculated by comparing the quantification of the relative proteins with ImageJ to that in control *Htt-Q16* animals, previously normalized to tubulin. We calculate the ratio using the 100 KDa isoform, since it is described as the ortholog of human p62 [41]. One-way ANOVA *p*-values: *** *p* < 0.001, ** *p* < 0.01, * *p* < 0.05 were calculated from two independent experiments of animals, and error bars indicate the standard deviations.

We then analyzed genetically whether the ability of *GS1* to ameliorate the motility in *Htt-Q93* animals was dependent upon a functional autophagic pathway. Using the RNAi technique, we reduced the expression of *Atg1/ULk1* or *Atg5*, key components of the macroautophagy pathway [39] in combination with the expression of *GS1*. This analysis showed that the ability of *GS1* to ameliorate the climbing defects of *Htt-Q93* animals was significantly reduced when *Atg1* or *Atg5* expression was reduced (Figure 5A, *Htt-Q93*, *GS1* vs., *Htt-Q93*, *GFP*; *Atg1-RNAi* ANOVA *p* < 0.0001; and *Htt-Q93*, *GS1* vs., *Htt-Q93*, *GFP*; *Atg5-RNAi* ANOVA *p* < 0.0001), suggesting that the rescue exerted by GS1 requires a functional autophagic pathway. The efficiency of the *Atg1* and *Atg5 RNAi* lines to reduce autophagy and the relative protein levels was tested (Supplementary Figure S9).

In autophagy, the maturation of the phagosome determines the amount of LC3 (Atg8a in *Drosophila*), that is recruited to the vesicles. This depends on the presence of p62/SQSTM1 (sequestosome1) (Ref(2)P in *Drosophila*) that is necessary for the recruitment of the ubiquitinated substrates to the autophagosomes [40,41]. The expression levels of Atg8a and Ref(2)P proteins are used in Western blot to mark a functional autophagic flux. Atg8a-I protein (15 kDa) is lipidated and cleaved to yield a fast-migrating band of approximately 7 kDa (Atg8a-II), while its direct interaction with Ref(2)P, by virtue of its ubiquitin-associated domain, results in Ref(2)P degradation. Our analysis showed that in the presence of *GS1* the amount of the fast-migrating Atg8a-II band significantly increased, and this was visible also when *GS1* was co-expressed with *Htt-Q93* (Figure 5B,C). Analysis of SQSTM1/Ref(2)P expression levels showed two distinct bands of approximately 100 and 62 KDa recognized by the *Drosophila* antibody, that were both significantly reduced in the presence of *GS1* (Figure 5B,C), and also when *GS1* was co-expressed with *Htt-Q93* (Figure 5B,C). All together these data support the hypothesis that GS1 promotes in neurons a small but active autophagic flux.

### 3.6. Expression of GS1 Reduces TOR Activation Visible by Phosphorylation of Its Substrate S6K

**Figure 6:**
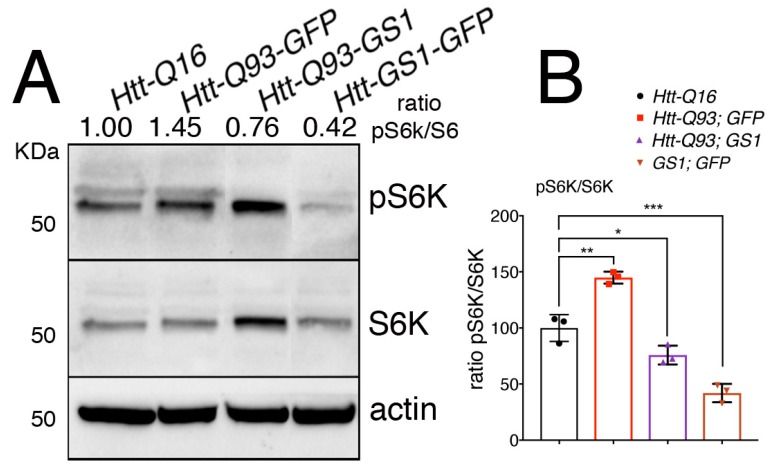
Expression of GS1 in neurons reduces S6K phosphorylation. (**A**) Western blot showing the level of phosphorylated S6K on Thr 398 and of S6K in lysates within 10 heads from animals expressing the indicated transgenes in neurons using the *elav-Gal4* driver. Actin was used as the loading control. (**B**) Graphic representing the ratio of S6K phosphorylation versus the unphosphorylated form of the protein. The ratio between pS6K/S6K was calculated from three independent experiments. One-way ANOVA *p*-values: *** *p* < 0.001, ** *p* < 0.01, * *p* < 0.05 were calculated from three independent experiments, error bars indicate the standard deviations.

When it is activated, TOR translocates onto the lysosomal membrane where it assembles in the TORC1 complex, resulting in its activation and phosphorylation of the downstream targets S6K and 4EBP [42]. To explore the ability of GS1 to affect TOR activation, we analyzed the level of phosphorylation of its target S6K on Thr-398, an event that is conserved also in *Drosophila* S6K [43] in lysates from the heads. This analysis revealed that expression of *UAS-GS1* in neurons significantly reduced the levels of S6K phosphorylation (Figure 6A,B, ANOVA *p* < 0.001), and a similar result was obtained when *UAS-GS1* was co-expressed with *UAS-Htt-Q93* (Figure 6A,B, ANOVA *p* < 0.05). Overall these new data suggest that GS1 activity could influence the level of essential amino acids involved in TOR activation, resulting in the inhibition of TOR complex1 and of the downstream phosphorylation of its targets including S6K.

### 3.7. Expression of GS1 in Neurons affects Glutamate and Glutamine Levels and Reduces Essential Amino Acids

**Figure 7:**
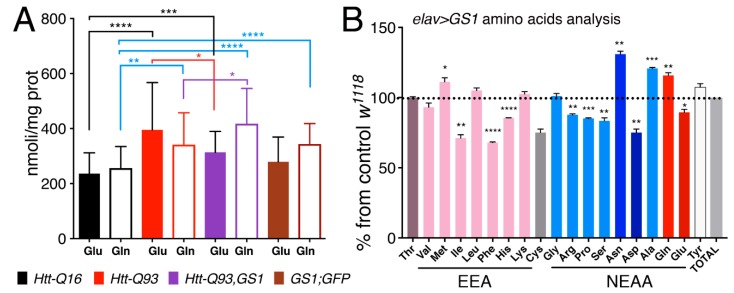
Expression of *GS1* in neurons reduces glutamate Glu and increases glutamine Gln, and influences amino acid levels. (**A**) Quantification of glutamate (Glu) and glutamine (Gln) from heads was analyzed by MS/HPLC in lysates from animals of the indicated genotypes. (**B**) Amino acids profiling from heads of animals expressing *GS1* in neurons; the data are indicated as the % of the amino acids concentrations in *GS1* versus the relative concentration in lysates from control *w^1118^* animals. 20 animals for each genotype were used. EAA = essential amino acids, NEAA = non-essential amino acids. The **** *p* < 0.0001, *** *p* < 0.001, ** *p* < 0.01, * *p* < 0.05 values were calculated from Student’s *t*-test from at least three independent experiments; the error bars indicate the standard deviations.

In order to test if expression of GS1 affected the level of glutamate (Glu) and glutamine (Gln) we next performed analysis of these amino acids, from the heads of our relative animals. This data revealed that in general glutamate levels are increased in the head of animals expressing *Htt-Q93* in neurons (Figure 7A, solid black vs. solid red columns, *t*-test *p* < 0.0001). Moreover, expression of *GS1* together with *Htt-Q93* significantly reduced this effect (Figure 7A, solid red vs. solid purple columns, *t*-test *p* < 0.05), and a small but significantly increase in Gln levels was still observed (Figure 7A, empty red vs. empty purple columns, *t*-test *p* < 0.05). Expression of *GS1* alone did not affect Glu concentration (Figure 7A solid black vs. brown columns, *t*-test ns) but significantly increased the levels of Gln (Figure 7A, empty black vs. empty brown columns, *t*-test *P*<0.0001). Suggesting that GS1 is able to convert Glu into Gln particularly when the levels of Gln are increased like in the presence of Htt-Q93.

To better identify the effect of GS1 on the level of expression of other amino acids, we analyzed the profile of Essential amino acids (EAA) and Non-Essential amino acids (NEAA) from the heads of animals expressing *GS1* in neurons, and compared it to that from control *Htt-Q16* flies (Figure 7B). These data confirmed that Gln concentration was significantly increased upon *GS1* expression (*p* < 0.01) and that Glu level was reduced (*p* < 0.05). In addition, EAA: isoleucine (Ile), phenylalanine (Phe), histidine (His) and NEAA:arginine (Arg), proline (Pro), serine (Ser) and aspartic acid (Asp) were also significantly reduced. Moreover, these data revealed the unexpected ability of GS1 to increase asparagine (Asn) level while reducing aspartic acid (Asp). This last observation, together with the increase of the Gln level, may suggest a novel role for this enzyme in neurons to activate asparagine synthetase (ASNS), an enzyme that hydrolyzes Gln to produce Asn from Asp and Glu. Notably, this enzyme was previously described in mammals as activated to bypass amino acid starvation and production of Asp necessary to sustain protein synthesis and cell survival [44].

### 3.8. Fibroblasts from Patients with Huntington’s Disease Have Impaired Autophagy and Reduced Level of GS1

**Figure 8:**
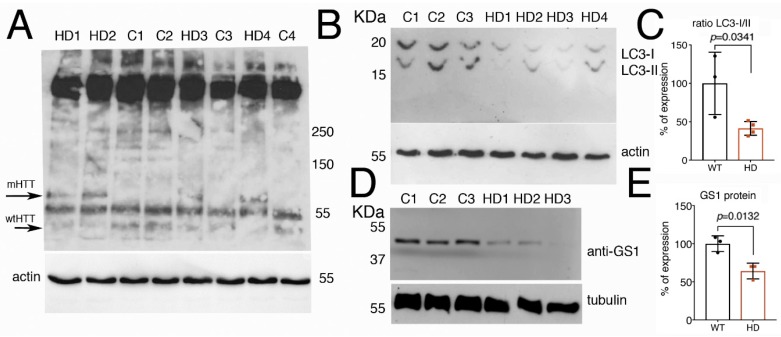
Fibroblasts from HD patients have reduced the expression of LC3 and GS1 proteins. (**A**) Western blot showing the pattern of expression of human Htt protein in fibroblasts from control healthy donors (C1–C3), with an average of 15–33 *CAGs,* or from HD patients (HD1–HD4) with an average of 78–90 *CAGs*. The anti-hHtt antibodies evidence the presence of a band of about 70 kDa (mHTT) that is visible only in lysates from cells deriving from HD patients, while in the lysates form controls, a band below 55 KDa is evident and preferentially expressed in healthy donors (wt-Htt). Experiments were repeated at least twice, data were analyzed from al least three different patients or controls, and a representative situation is shown. (**B**) Western blot showing the cleavage of LC3 protein in the lysates of fibroblasts from control healthy donors and from HD patients. The fast-migrating form of LC3-II (upper panel) is significantly reduced in the extracts from fibroblasts of HD patient as compared to lysates from control donors. Actin is shown as a control for loading (lower panel). (**C**) Quantification of the ratio LC3-II/I from (**B**). (**D**) Western blot showing that expression of GS1 in fibroblasts from HD patients is reduced compared to its expression in control donors, tubulin was used as a control for loading. (**E**) relative quantification analysis of GS1 in fibroblasts from (**D**). The *p* values were calculated from Student’s *t*-test from the relative number of samples.

Since autophagy is known to be defective in HD [45,46], we analyzed whether the autophagic flux was compromised in fibroblasts derived from patients carrying different lengths of the *(CAG)s* in the *exon1*-of the *Huntingtin* gene (*Htt*). Analysis by Western blot on the level of hHtt protein expression in these cells (Figure 8A) showed the presence in all the samples of a band running above 250 kDa, possibly the full-length Htt protein with the predicted size of 350 KDa [47]. In addition, we observed a band of about 70 kDa only present in lysates from cells derived from patients carrying in the *Htt* gene a sequence of 78–90 *CAG* repeats (mHTT). A band of about 55 KDa was present in all the samples but more evident in lysates from fibroblasts from healthy donors carrying a sequence of about 17–33 *CAG* repeats in the *Htt* gene (wt-Htt). The human huntingtin protein is cleaved by calpain at around amino acids 437–586 to generate a series of proteolytic fragments of about 50 KDa [48], we believe that the difference in the molecular weight observed in the western blot of the HTT proteins, is due to the presence of a the CAGs sequence in the proteolytic fragments of the mHTT that runs at about 70 KDa, as compared to 50 KDa in extracts from fibroblasts from healthy donors, that is recognized by the anti-*N*-terminus Htt antibodies.

The proteolytic cleavage of LC3 is considered a hallmark of autophagy, our analysis showed that in cells from HD patients, the fast-migrating bands, corresponding to the cleaved LC3-II form, is significantly reduced compared to its level in fibroblasts from control healthy donors (Figure 8B,C, Student *t*-test *p*-0.0341), suggesting that the autophagic flux has been compromised in HD cells. In addition, we analyzed the level of GS1 protein expression by western blot, and to our surprise, we found that GS1 protein was 45% reduced in fibroblasts from patients, as compared to that in fibroblasts from control donors (Figure 8D,E, Student *t*-test *p* = 0.0132). Taken together, these data from human cells corroborate our results described in flies that suggest a potential novel conserved function for GS1 to control autophagy, a function that may be impaired in pathological conditions such as HD.

## 4. Discussion

The glutamate–glutamine cycle (GGC) is a sequence of biochemical events between neurons and glia necessary for the control of glutamate homeostasis. Previous studies showed that this cycle is impaired in mouse and *Drosophila* models for HD [49,50]. In addition, the activity of Glutamine Synthetase 1 (GS1), a key regulator of the GGC, was found to have been reduced in post mortem brains of HD patients [51,52]. To elucidate the role of GS1 in HD, we modulated its activity in neurons using a *Drosophila* model expressing the mutated human exon1 *Htt-Q93* that mimics many aspects of the neurodegeneration induced by toxic polyglutamine expansion in HD patients [34]. Our data show that neuronal expression of the *Drosophila* homolog of human *GS1* ameliorates animal motility and reduces neuronal death induced by the expression of *Htt-Q93*.

Moreover, we found that co-expressing *GS1* with *Htt-Q93* reduces the size of the toxic Htt-Q93 aggregates, and increases the levels of macro-autophagy, herein referred to as autophagy, establishing a novel link between the GGC and functional autophagy in post-mitotic cells.

Autophagy has a well-established beneficial role in several processes relevant for neurons under both normal and pathological conditions [6,53]. It is a conserved mechanism controlled by key proteins like Atg1/ULK1, a kinase essential for the initial formation of the complex, then Atg5, that is involved together with LC3/Atg8a in the autophagosome maturation and lipidation of LC3/Atg8a to its proteolytic cleavage to Atg8-II. p62/SQSTM1/Ref(2)P binds to the autophagosome-coating protein LC3/Atg8a and activates the fusion of the phagosome with the lysosome to degrade its cargo [21]. Polymorphisms in the *ATG* genes, including *ATG5* and *ATG7*, have been associated with an early onset of neurodegenerative diseases, including HD [54]. Moreover, the important role of autophagy in neurons was revealed by the conditional knock-out of *ATG5* and *ATG7* that results in progressive motoneuron defects, alteration in autophagosome formation and accumulation of ubiquitin-positive protein aggregates in degenerating neurons [55,56,57]. These phenotypes are conserved in *Drosophila*, where *atg7D77* mutants have a short life-span, reduced motility and the accumulation of ubiquitinated proteins [58].

Our data show that the ability of GS1 to ameliorate the motility of *Htt-Q93* flies depends on a functional autophagic pathway since the reduction of *atg1* and *atg5* completely abolished the rescue by GS1. Moreover, the presence of GS1 in neurons enhances the cleavage/lipidation of Atg8 (Atg8-II) and reduces the level of Ref(2)P/SQSTM1, suggesting an increase of the autophagic flux in neurons. Autophagy is inhibited by nutrients and amino acids that induce TORC1 activation [10,21]. Stimulation of TORC1 at the lysosome membrane depends on the ability of amino acids to activate the Rag(s) (GTPases), the Ragulator complex and the vacuolar H^+^ adenosine triphosphatase (v-ATPase) [59]. However, TOR activity is controlled specifically by certain branches of amino acids [60], most relevantly, glutamine [61,62], leucine [42,61,63], and arginine [64].

Our data show that expression of GS1 reduces the phosphorylation of the S6K protein, a target of TOR complex 1, thereby suggesting that it plays a role in the regulation of its activity. GS1 converts glutamate (Glu) into glutamine (Gln), and amino acids analysis shows that expression of *GS1* alone or in the presence of *Htt*-Q93 increases the level of Gln (Figure 8B). Gln is involved in a multiplicity of metabolic pathways, and its function in TOR signaling still presents some controversial results. Gln in epithelial cells binds to the adenosine diphosphate ribosylation factor-1 GTPase (Arf1) to directly activate TORC1 at the lysosome membrane [60]. Our data in neurons however show that in the presence of GS1, TOR signaling is reduced even if the glutamine level increases, so this may suggest that in terminally-differentiated cells, GS1 induces a “starvation-like” condition that favors autophagy without the activation of TORC1. In support of this mechanism, van der Vos and colleagues showed that in cancer cells under nutrient starvation, transcriptional induction of GS1 by FOXO3 resulted in autophagy and survival with the concomitant inhibition of the TOR pathway [65]. We do not know at the moment if this pathway may be responsible for any GS1 activation in neurons, but we found that the expression of *Drosophila* FOXO transcriptionally induces *dGS1-mRNA*, suggesting that this regulation is conserved also in flies (Supplementary Figure S5).

Expression of GS1 also reduces the level of Glu, a nonessential amino acid that functions as excitatory neurotransmitter in the central nervous system, and acts as a substrate in many distinct biochemical reactions [66]. Glu is synthesized from Gln, α- ketoglutarate and 5-oxoproline as a precursor for the biosynthesis of various amino acids including proline (Pro) and arginine (Arg) [67,68]. Interestingly, in the heads of animals expressing GS1, we found reduced levels of Pro and Arg, both relevant amino acids in the mechanism of TOR activation through specific amino acid sensors [59]. In particular, the lysosomal amino acid transporter SLC38A9 was proposed relevant for TOR activation by Arg in mammalian epithelial cells [69]. So far, there are no data describing the mechanism of activation by SLC38A9 in neurons. However, since we found Arg levels reduced in the heads of animals expressing GS1, while Gln levels were high, we speculate that Arg activation of SLC38A9 in neurons may act downstream of Gln in the stimulation of TOR pathway, since Gln is not a limit factor.

It is worthy to note that, although the *Drosophila* SLC38A9 transporter has not been characterized yet, the *Drosophila* genome contains three genes expressed in the brain (CG13743, CG30394 and CG320181) [70,71,72], whose sequences are significantly homologous to that of human SLC38A9 [73], suggesting that they may encode for the fly functional homolog(s) of SLC38A9.

Our data also show that GS1 increases the level of Asn while the level of Asp decreased, suggesting an upregulation of asparagine synthetase (ASNS), a transaminase that is induced during nutrient starvation to indirectly synthetize amino acids (Asn) to sustain protein synthesis [44,69,74,75]. Similarly, we saw the levels of alanine (Ala) increased, suggesting the activation also of alanine synthetase, another transaminase, whose level of activation may be stimulated when glutaminolysis is higher to produce Glu from Gln to support metabolic pathways of the biosynthesis of proteins [76].

Finally, we analyzed autophagy in human fibroblasts derived from healthy donors or patients with HD. These experiments revealed that fibroblasts carrying the *mHtt* suffer of a defect in the expression and possible cleavage of the LC3 gene/protein, suggesting that autophagy is impaired in these cells. These data allow to postulate that autophagy may be defective also in post-mitotic, primary neurons from HD patients; in the near future we would like to carry out experiments to validate this hypothesis in neuronal cells derived from iPSCs from donors or from patients carrying different CAGs mutations in the *huntingtin* gene.

In summary, malfunctions in the components of the glutamate–glutamine cycle have been detected in many neurodegenerative diseases, including Huntington disease [51,77,78]. Here we show a novel function for GS1, a member of the GGC, to control autophagy in post-mitotic cells, like neurons that are highly dependent on the endo–lysosomal pathway for their functional homeostasis. The function of GS1 in neurons may be important for the control of essential amino acids, including the switch to increase Asn, indicating a role in the biochemical homeostasis of neurons that maintain autophagy active. Here we can speculate that this function for GS1 in controlling autophagy is conserved also in humans, as fibroblasts from HD patients have defective autophagy due to the reduced level of LC3 accompanied by a reduction in GS1 levels.

## Supplementary Materials

The following are available online at https://www.mdpi.com/2073-4409/9/1/196/s1; Supplementary Figure S1: *ClustalW* amino-acid sequence alignment of *Drosophila* GS1 (UniProt E1JHQ1) and of human GLUL (UniProt P15104); Supplementary Figure S2: Reduction of GS1 enhances retinal defects induced by Htt-Q93; Supplementary Figure S3: Expression of *GS1* rescues neuronal death induced by *Htt-Q93*; Supplementary Figure S4: Schematic representation of the N-terminus region of the huntingtin protein and time line of the advent of the motility defects induced by Htt-Q93 expression; Supplementary Figure S5: Expression of GS1 induces *FOXO-mRNA*; Supplementary Figure S6: In adult males, co-expression of *GS1* with *Htt-Q93* in neurons partially rescues animal survival (A) but not climbing defects (B) induced by *elav^c155^-Htt-Q93* in adult males; Supplementary Figure S7: MSO (methionine sulfoximine) specifically inhibits GS1 activity in lysates from heads of *elav^c155^*/+ *and elav^c155^*/+; *EP GS1^G3347^* females; Supplementary Figure S8: Co-expression of *GS1* with *Htt-Q93* in neurons partially rescues the climbing defects induced by expression of *elav^c155^-Htt-Q93* visible in adult females, using two others independent recombinant *Htt-Q93*, *GS1* lines; Supplementary Figure S9: Efficiency of *Atg1-RNAi* and *Atg5-IR 24-1* in reducing autophagic puncta and the relative protein expression in adult heads. Supplementary Figure S10: Expression of *elav-Atg8-mCherry* in the calyx of third instar larval brains. Supplementary Excel-file 1: ANOVA analysis; Supplementary Video S1: larvae *elav-Htt-Q16*; Supplementary Video S2: larvae *elav-Htt-Q93*; Supplementary Video S3: adult climbing *elav-Htt-Q93* and *elav-Htt-Q16*.
