# Supplementary material for: Glutamine Synthetase 1 Increases Autophagy Lysosomal Degradation of Mutant Huntingtin Aggregates in Neurons, Ameliorating Motility in a Drosophila Model for Huntington’s Disease
Source: Cells. 2020 Jan 13;9(1):196. doi: 10.3390/cells9010196 (PMC7016901; doi:10.3390/cells9010196)
Supplement: Supplementary file 1 [file cells-09-00196-s001.zip › Supplementary Files Vernizzi revised Cells 12:01:20/Supplementary Vernizzi et al, Cells.docx]

**Supplementary Figure S1:**

***ClustalW* amino-acid sequence alignment of *Drosophila* GS1 (UniProt E1JHQ1)**

**and of human GLUL (UniProt P15104).**

***
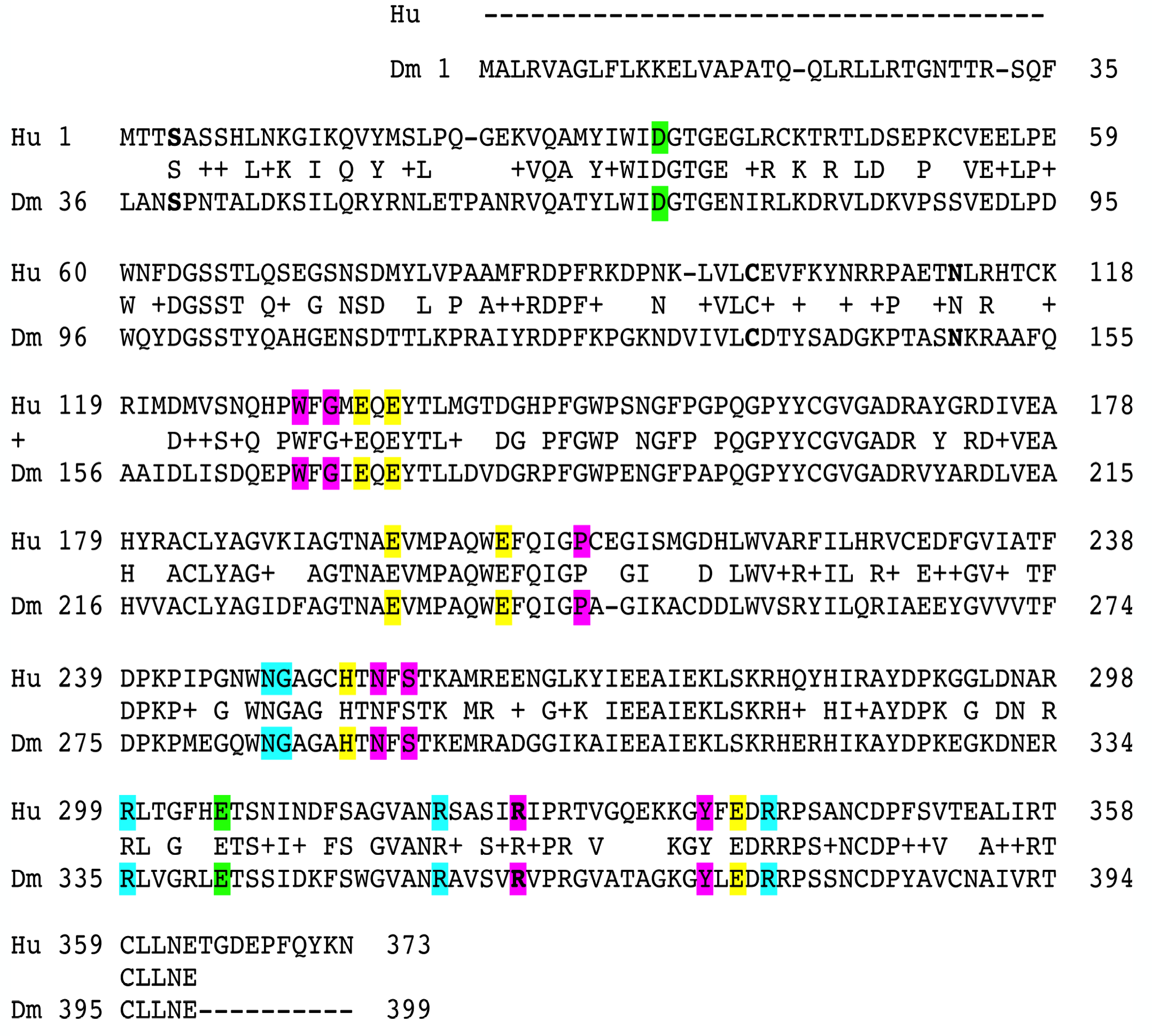
***

The two proteins show 61 % identity in the whole sequence that increases to 70.5 % on 298 aminoacids. The common ligand binding residues are indicated with purple (ATP), blue (glutamate) green (ammonia), yellow (metal coordination) [[1](#_ENREF_1)].

**Supplementary Figure S2:**

**Reduction of *GS1,* using RNAi techniques, enhances the retinal defects induced by expression of *Htt-Q93.***

Photographs of eye from adult females at the indicated days after eclosion (DAE), expressing *UAS-Htt-Q93 alone* (upper panels) or together with *UAS-GS1-RNAi* (lower panels) using the retina-specific *GMR-Gal4* promoter. These data show that co-expression of *GS1-RNAi* enhances the retinal degeneration phenotype induced by the expression of *Htt-Q93* that at 8 DAE (E) is comparable to that of *UAS-Htt-Q93* animals at 20 DAE (C).

**
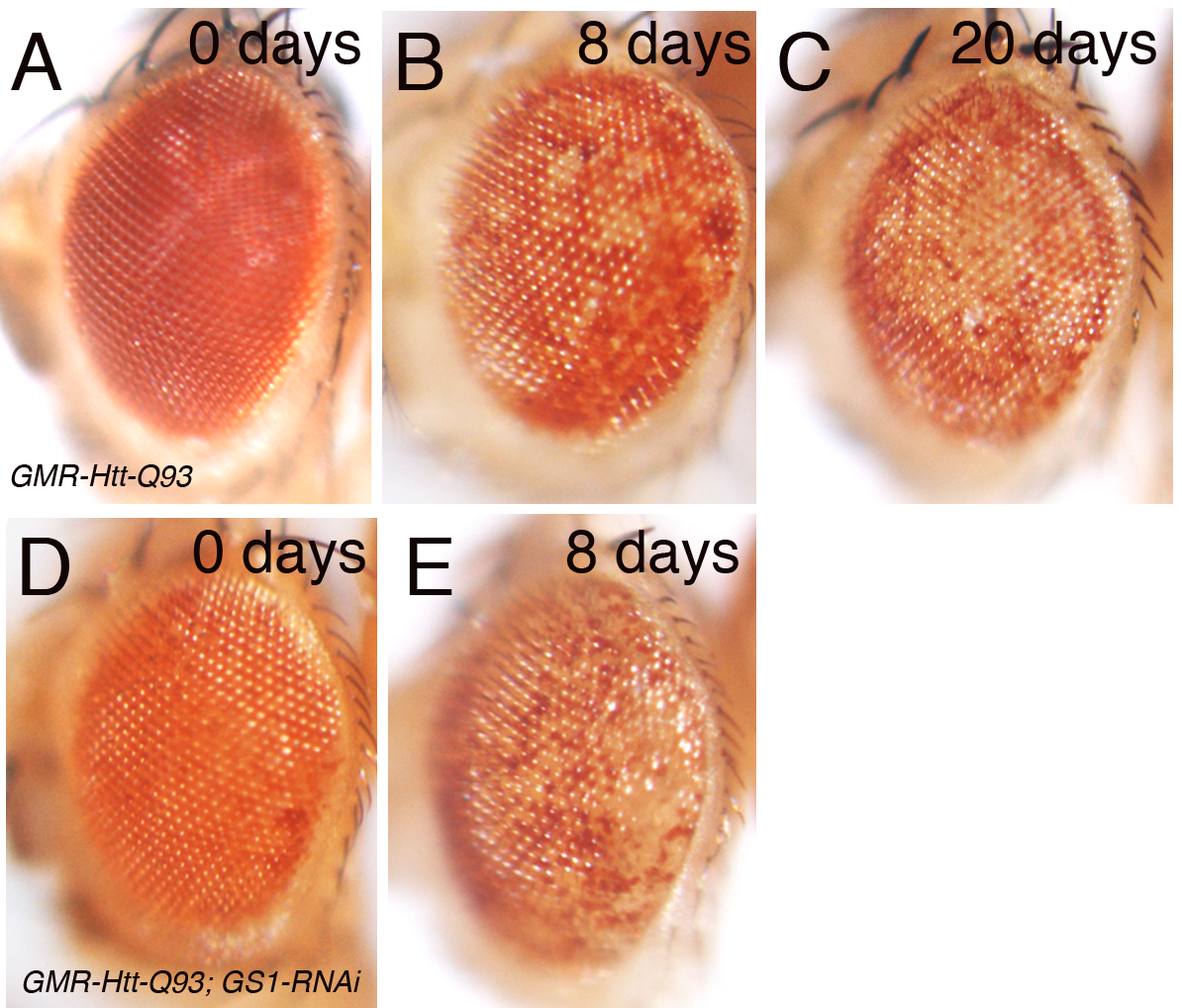
**

**Supplementary Figure S3:**

**Expression of *GS1* rescues the reduction of GFP induced by expression of *Htt-Q93* in neurons.**

Cell death can be visualized and quantified in the adult eyes by measuring the decrease of the fluorescence of GFP expressed by *UAS-GFP* together with mutants of *huntingtin* that is known to induce cell death [[2](#_ENREF_2), [3](#_ENREF_3)]. Using the *GMR-Gal4* promoter we expressed *UAS-GFP* (*GMR>GFP*) together with *UAS-HTTQ93*. The fluorescence in the eyes of adult females was acquired in animals at 20 days after eclosion (DAE). (A-D) Representative photographs showing the lateral view of eyes co-expressing GFP with *Htt-Q16* as a control (A), *Htt-Q93* (B), *Htt-Q93* together with *GS1* (C) and *GS1* alone. In these experiments is visible that the expression of GS1 (C) rescues the reduction of GFP induce by *Htt-Q93* (C). Quantification of GFP was measured from images using the program *imageJ* and calculated as Integrated Density in a fix area of 20 ommatidia in the center of the compound eye. A minimum of four animals for each genotype were used and experiments were repeated twice.

**
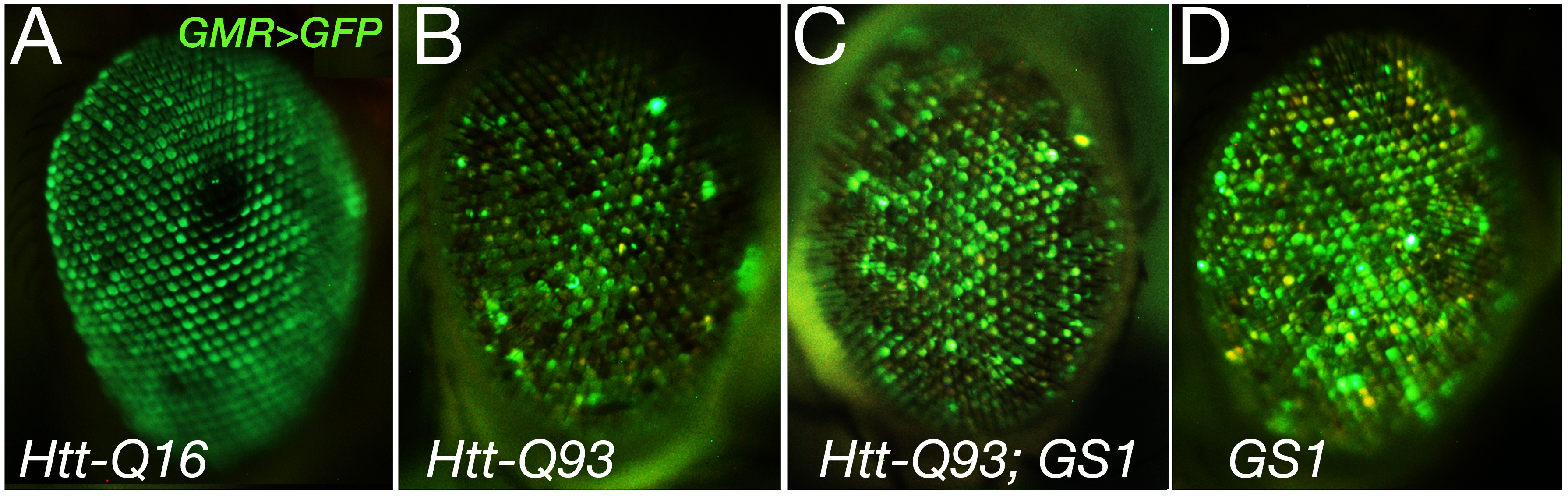
**

**Supplementary Figure S4:**

(A) Schematic representation of the N-terminus region of the huntingtin protein showing the position of the expanded CAGs sequence (black). (B) Time line of the advent of the motility defects caused by the expression of the *UAS-Htt-Q93* using the *elav-Gal4* promoter; of note that motor defects and the presence of Htt-Q93 aggregates are already detected after 24-48 hours after egg laying (AEL). (B) Time line of the developmental defects observed in the elav-Htt-Q93 animals.

E =embryos, L1, L2 and L3 = first, second and third larval instar respectively. (C) Petri dish used for the motility assay of larvae (arena). A grid was drawn on the Petri dish containing 1% agarose in 1X PBS, the red spot is where each larva was posed at the beginning of the test. The number of lines crossed by each larva within 1 minute was scored. (D-F) Movies showing the motility of *elav-Htt-Q16* (D, Supplementary movie 1) and *elav-Htt-Q93* (E, Supplementary movie 2) 3^rd^ instar larvae. (F) Movie showing an adult climbing assay using *elav-Htt-Q16* and *elav-Htt-Q93* females at 15 DAE (Supplementary movie 3).


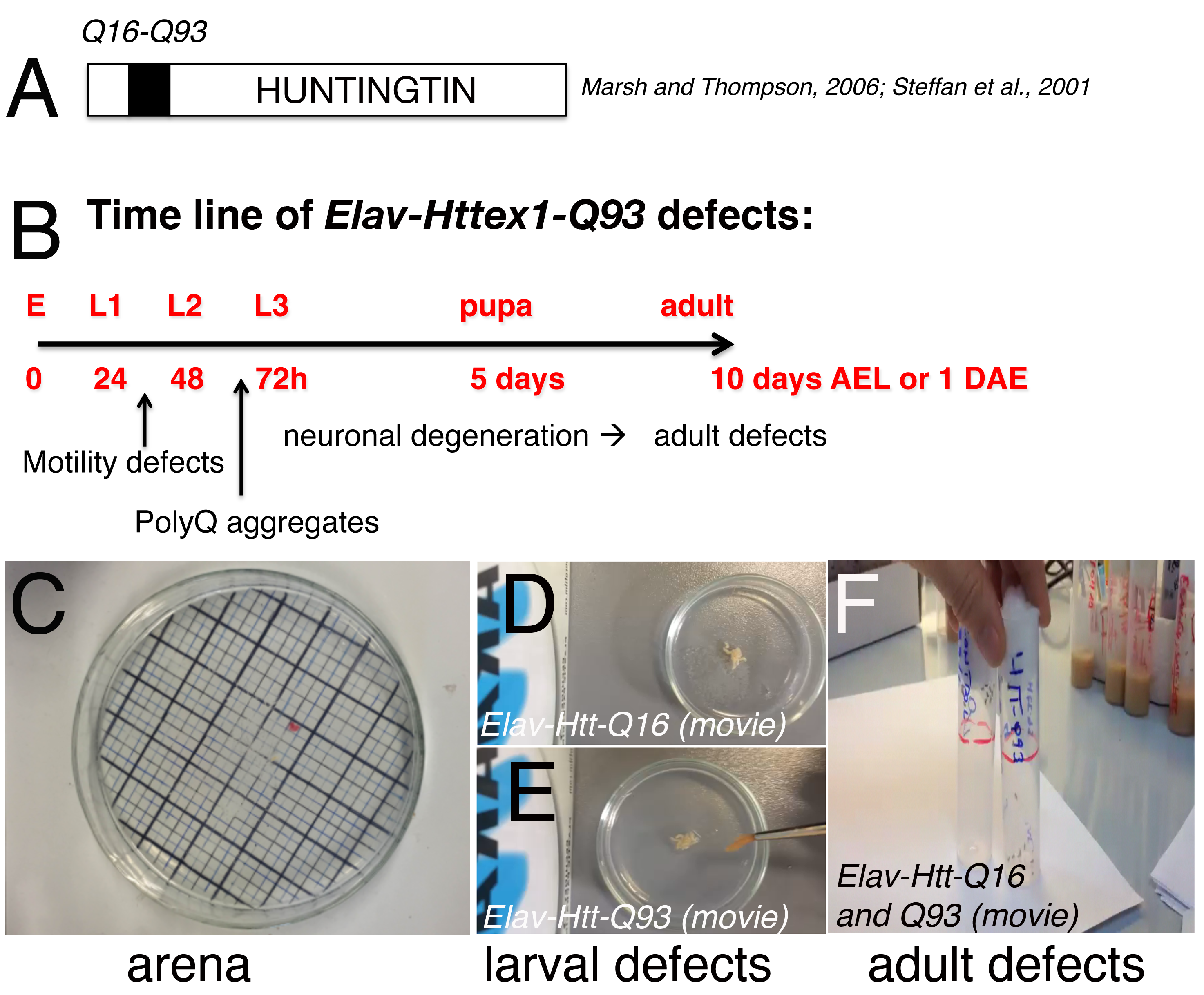


**Supplementary Figure S5:**

**Expression of GS1 induces *FOXO-mRNA*.**

Quantitative RT-PCR of *GS1-mRNA* in whole larvae from 3dr instar animals expressing the indicate *UAS-*using the *actin-Gal4* ubiquitous driver. *actin5C* was used as the internal control. **P*<0.05, *****P*<0.0001 values were calculated from Student’s *t-*test from at least three independent experiments, error bars indicate the standard deviations.

**Supplementary Figure S6:**

**In adult males, co-expression of *GS1* with *Httex1-Q93* in neurons partially rescues animal survival (A) but not climbing defects (B) induced by *elav^c155^-Httex1-Q93*.**

Lethality in adult males was scored over time. Males expressing *Httex1-Q93* (red) showed 50% of lethality at about 4-5 DAE, while their climbing activity was significantly reduced with 50% reduction at about 2-3 DAE, compared to control *Httex1-Q16* males (black) (ANOVA *P*<0.0001). Co-expression of GS1 was able to partially rescue these defects, as shown in *Httex1-Q93,GS1* (purple) one-way ANOVA analysis between *Httex1-Q93* and *Httex1-Q93,GS1* was significant only for the survival ret showed (ANOVA *P*<0.05). Expression of *GS1* alone (brown) was comparable to control *Httex1-Q16* animals (black) and did not show any significant defects.


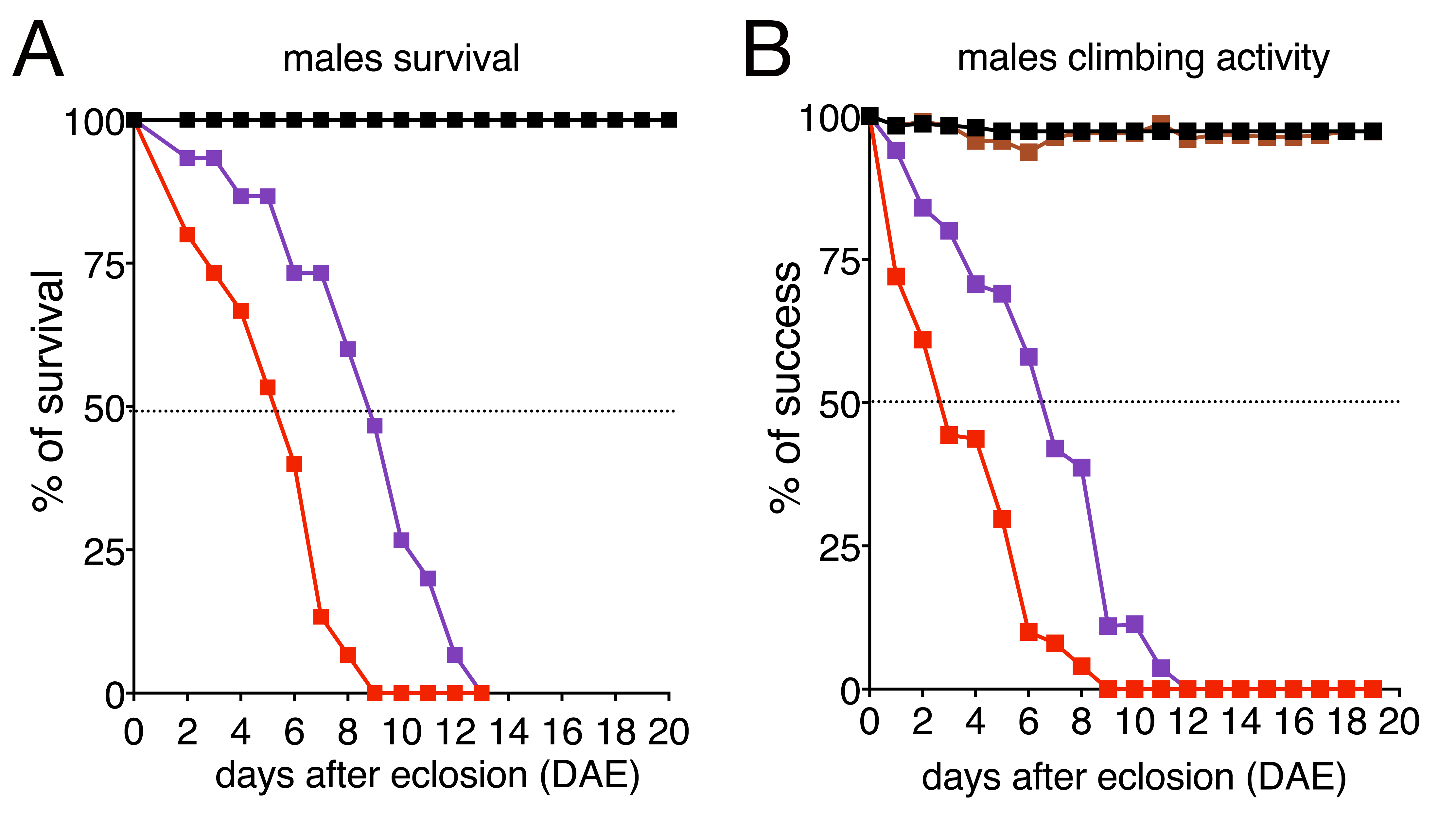


**Supplementary Figure S7:**

**MSO (methionine sulfoximine) specifically inhibits GS1 activity in lysates from heads of *elav^c155^*/+ *and elav^c155^*/+; *E{P} GS1^G3347^* females.**

Lysates from the heads of females of the indicated genotype were treated with the specific GS1 inhibitor MSO at 1mM final concentration prior the analysis of GS1 enzymatic activity, as described in material and methods.

**
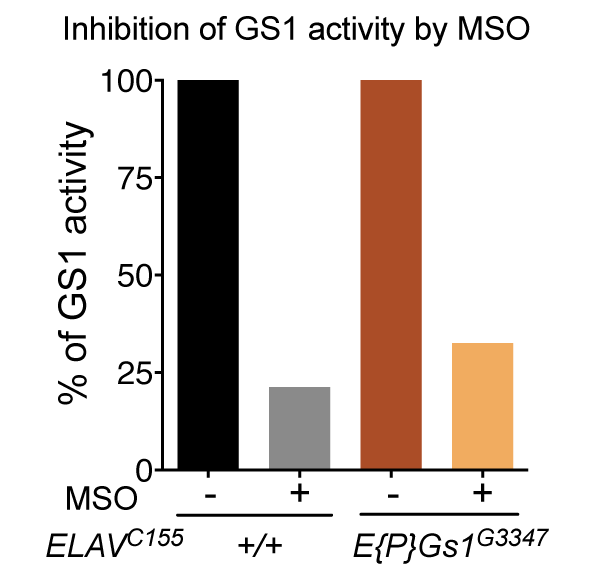
**

**Supplementary Figure S8:**

**Co-expression of *GS1* with *Htt-Q93* in neurons partially rescues the climbing defects induced by expression of *elav^c155^-Htt-Q93* visible in adult females, using two others independent recombinant *Htt-Q93*, *GS1* lines.**

We analyzed the ability of three independent lines, generated by recombination of *UAS-GS1 with UAS-Htt-Q93* to rescue the motility defects induced by *Htt-Q93* expression. ANOVA *P-*values: *Httex1-Q16 vs. Httex1-Q93* *P*<0.0001. Expression of *GS1* ameliorate these defects as seen using three independent *Httex1-Q93, GS1 lines:*

Line 1: *Httex1-Q93* vs. *Htt-Q93, GS1* (1), (ANOVA, *P*<0.05)

Line 3: *Httex1-Q93* vs. *Httex1-Q93, GS1* (3), (ANOVA *P*<0.01)

Line 4: *Httex1-Q93* vs. *Htt-Q93, GS1* (4)*,* (ANOVA*, P*<0.05)

For complete ANOVA analysis see supplementary Excel file.

**
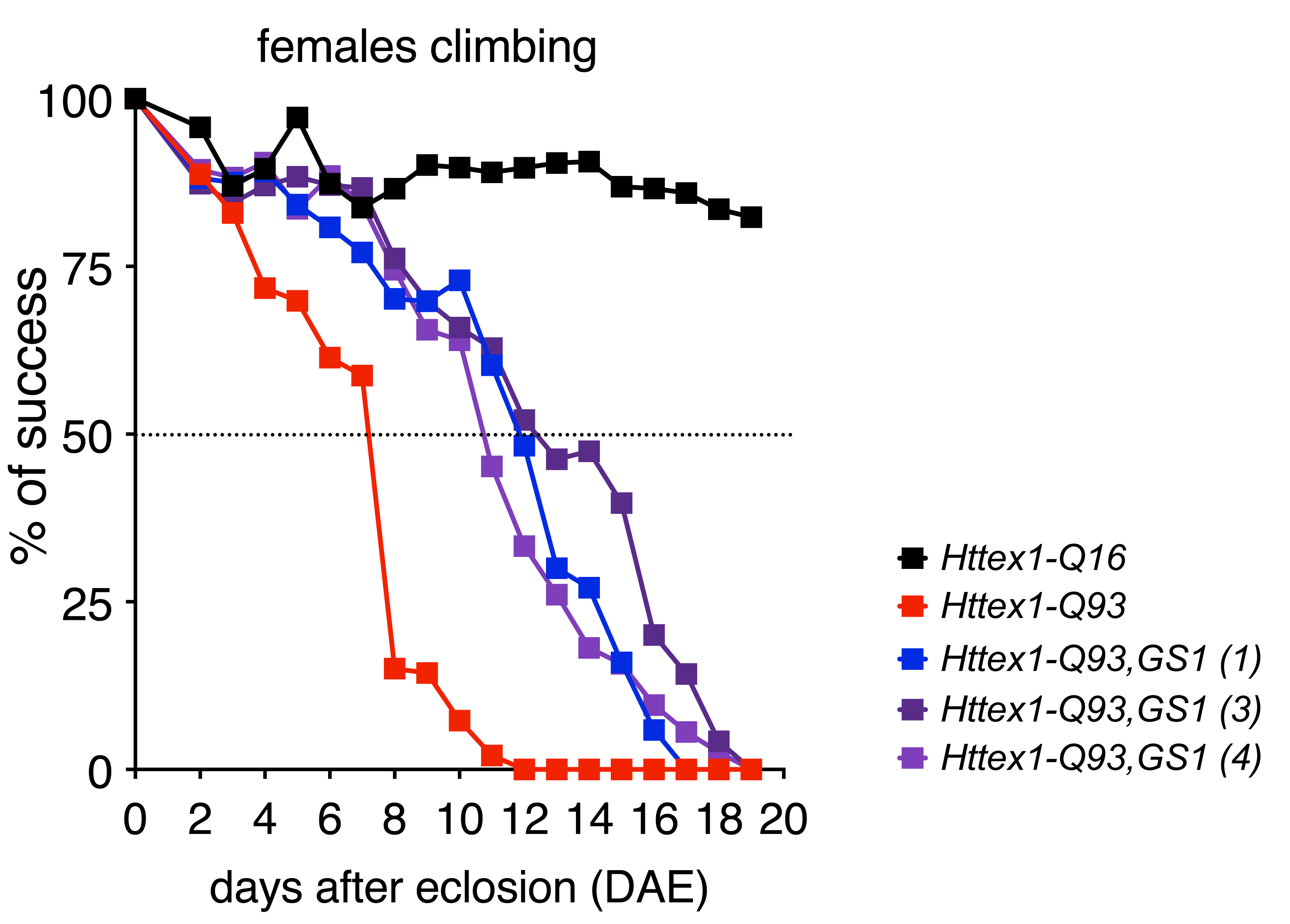
**

**Supplementary Figure S9: Efficiency of *Atg1-RNAi* and *Atg5-IR 24-1* in reducing the relative protein expression in adult heads.**

(A-D) Reduction of *Atg1-mRNA* decreases endogenous autophagy in neurons. Quantification of autophagic puncta, from confocal images of brains from third instar larvae expressing *elav-Gal4; mCherryAtg8a* in the neurons of the calyx that marks autophagosomes. (A) Integrated density of autophagic puncta, corresponding to autophagosome density (see materials and methods) of mCherry-Atg8a was measured in the calyx from: control animals *elav>w^1118^* (A black bar), or animals expressing *UAS-Atg1-RNAi* using two different transgenes on two different chromosome: *elav>mCherryAtg8a-RNAi*: on the II chromosome (A, gray bar) or on the III chromosome (A, dark gray bar). This analysis shows a significant decrease of Atg8a puncta by *Atg8a-RNAi* expression, while there is no significant difference in Atg8-mCherry levels between samples expressing *Atg1-RNAi* (II) or *Atg1-RNAi (III)*. (One-way ANOVA test, error bars SEM, ** *P*< 0,01; *** *P*< 0,001). (B-D) Confocal photographs of the region of the calyx from animals of the indicated genotypes where the analysis in A was performed. (E) Western blots showing the ability of RNAi line to reduce the expression of ATG1 (left) and ATG5 (right) proteins using the promoter *repo-Gal4* in lysates from the heads of females. Repo is a promoter for glia and we use it as a control for the efficiency of *Atg1-Rnai* and *Atg5-RNAi* lines in a set of experiments involving glia. Here we used it here to show the efficiency of our lines. Tubulin was used as loading control.


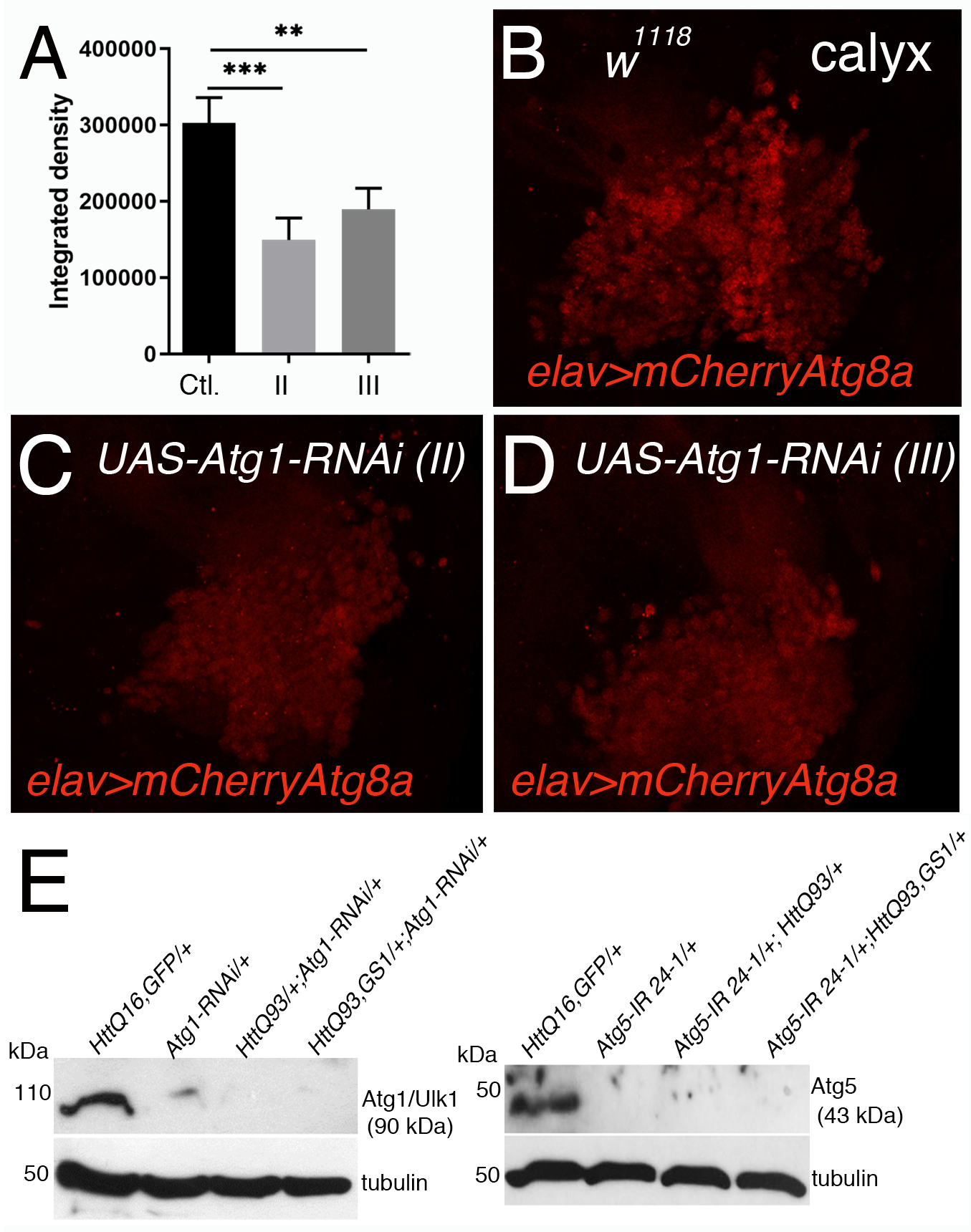


**Supplementary Figure S10: Expression of *elav-Atg8-mCherry* in the calyx of third instar larval brains.** Higher magnification of the confocal pictures of Figure 4 (E-H) showing the staining of autophagosome visible as autophagic puncta in the calyx of animals of the indicated genotype expressing *Atg8-mCherry* using the elav-Gal4 promoter.


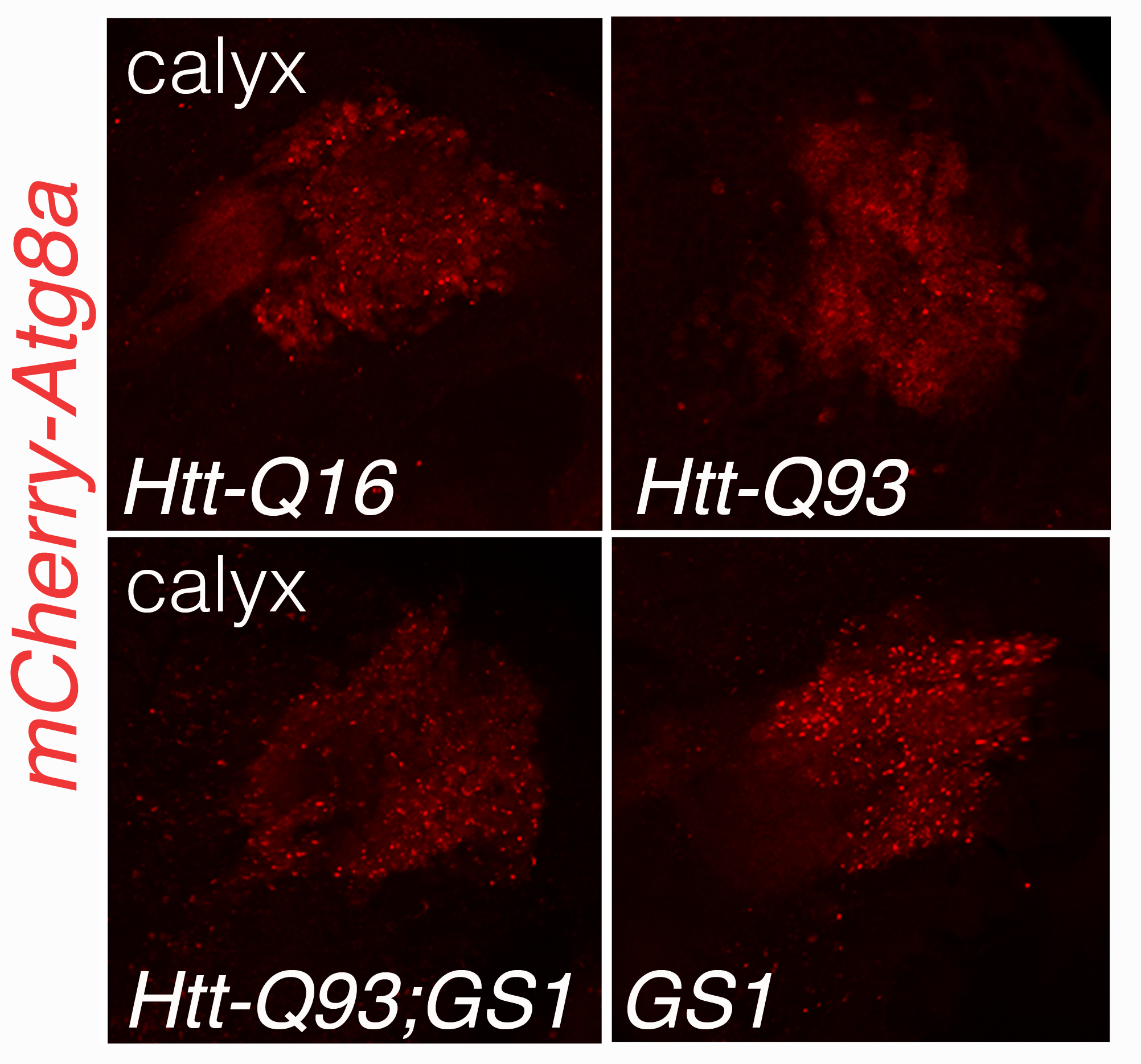


**References:**

1. Torreira E, Seabra AR, Marriott H, Zhou M, Llorca O, Robinson CV, Carvalho HG, Fernandez-Tornero C, Pereira PJ: **The structures of cytosolic and plastid-located glutamine synthetases from Medicago truncatula reveal a common and dynamic architecture**. *Acta crystallographica Section D, Biological crystallography* 2014, **70**(Pt 4):981-993.

2. Zhang S, Binari R, Zhou R, Perrimon N: **A genomewide RNA interference screen for modifiers of aggregates formation by mutant Huntingtin in Drosophila**. *Genetics* 2010, **184**(4):1165-1179.

3. Lievens JC, Rival T, Iche M, Chneiweiss H, Birman S: **Expanded polyglutamine peptides disrupt EGF receptor signaling and glutamate transporter expression in Drosophila**. *Human molecular genetics* 2005, **14**(5):713-724.
